# Supplementary figures and images for: Unraveling the role of host kinase PIM1 in Toxoplasma gondii infection: Implications for therapies
Source: PLoS Negl Trop Dis. 2026 Jan 20;20(1):e0013915. doi: 10.1371/journal.pntd.0013915 (PMC12844532; doi:10.1371/journal.pntd.0013915)

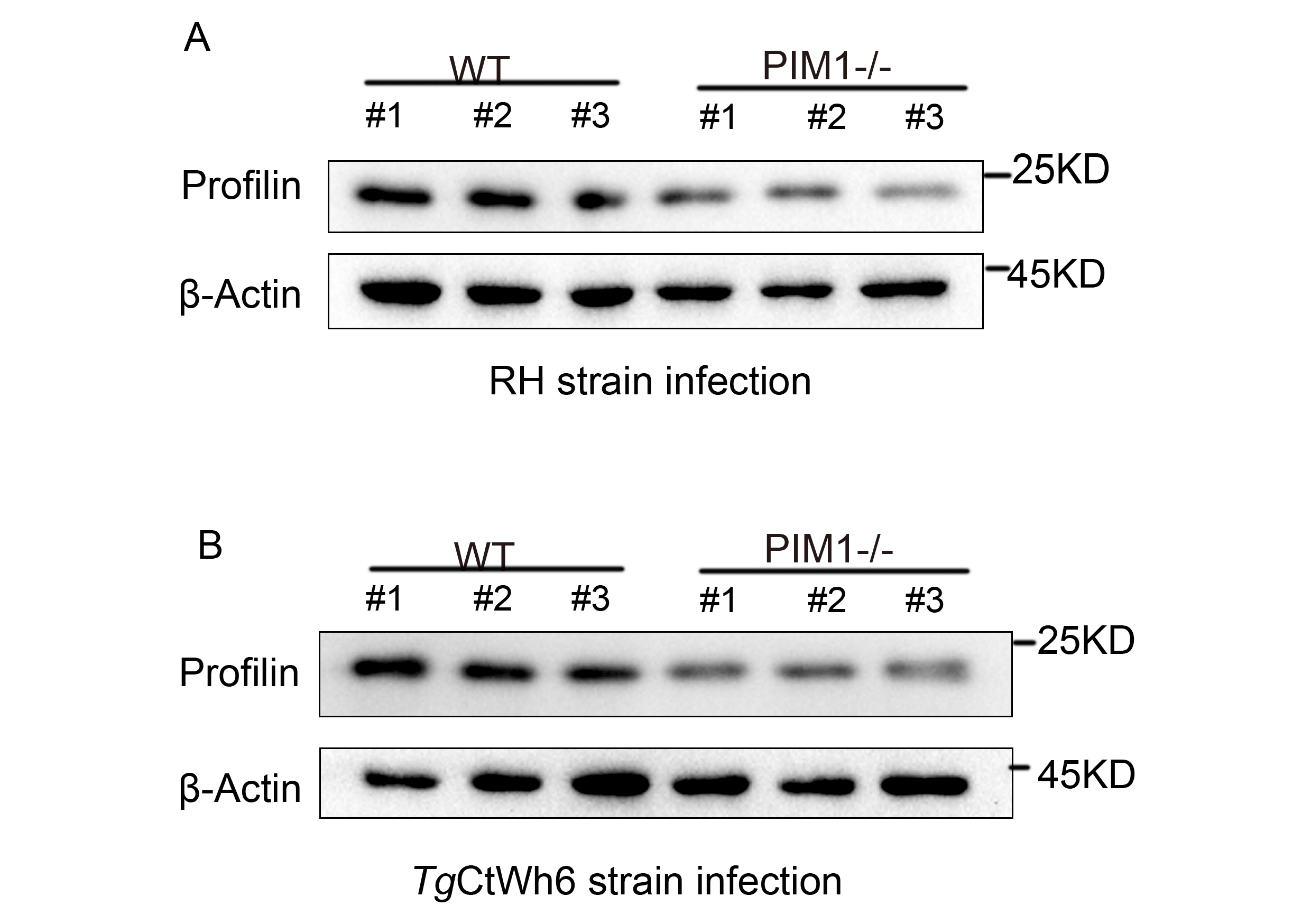

Supplement: S1 Fig — Depletion of PIM1 dampens the proliferation of the RH strain and the Chinese 1 genotype Wh6 strain of T.gondii. In HeLa WT and PIM1-/- cells, WB showing parasite profilin (upper band) and loading control β-actin (lower band) after T. gondii infection at MOI = 3 for 24 hours. Each blot contains three lanes (#1, #2, #3) corresponding to three independent biological replicates. (TIF) [file pntd.0013915.s001.tif]

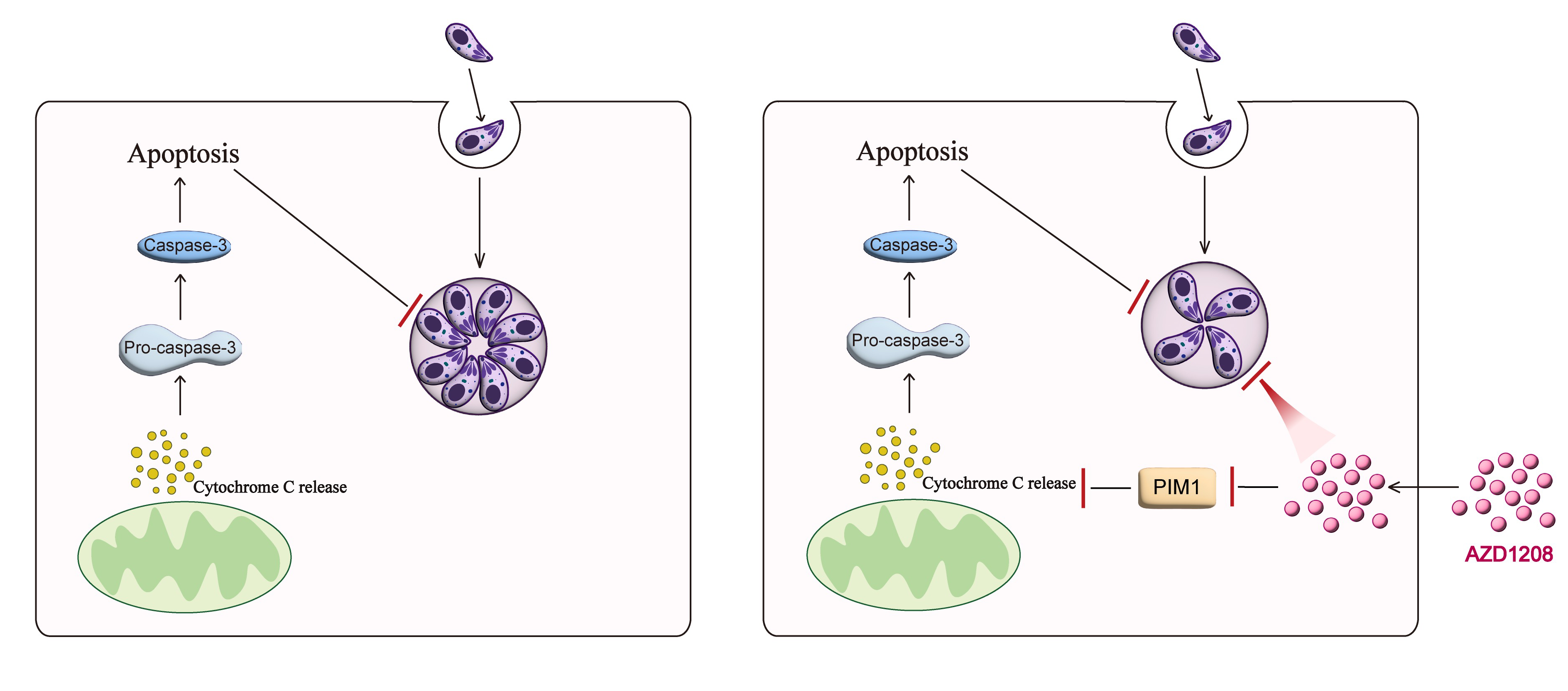

Supplement: S2 Fig — The character of PIM1 in regulating proliferation of T. gondii. PIM1 facilitated the proliferation of T. gondii via suppressing apoptosis. AZD1208, a small molecule inhibitor of PIM1, led to the elimination of T. gondii and consequently reduced the parasite load. (TIF) [file pntd.0013915.s002.tif]
