# Supplementary material for: Unraveling the role of host kinase PIM1 in Toxoplasma gondii infection: Implications for therapies
Source: PLoS Negl Trop Dis. 2026 Jan 20;20(1):e0013915. doi: 10.1371/journal.pntd.0013915 (PMC12844532; doi:10.1371/journal.pntd.0013915)
Supplement: S1 Table — (DOCX) [file pntd.0013915.s003.docx]

Table 1. Primers used in the article.

| Primer Name | Sequence(5’→3’) |
| --- | --- |
| hGAPDH forward | 5ʹ- ATGACATCAAGAAGGTGGTG-3ʹ |
| hGAPDH reverse | 5ʹ- CATACCAGGAAATGAGCTTG-3ʹ |
| TgITS1 forward | 5ʹ- AATATTGGAAGCCAGT GCAGG-3ʹ |
| TgITS1 reverse | 5ʹ- CAATCTTTCACTCTCTCTCAA-3ʹ |
| mGAPDH forward | 5ʹ- ATGGTGAAGGTCGGTGTGAA-3ʹ |
| mGAPDH reverse | 5ʹ- CGCTCC TGGAAGATGGTGAT-3ʹ |
| mPIM1 forward | 5ʹ- TACACGGACTTTGATGGG ACC 3ʹ |
| mPIM1 reverse | 5ʹ- CTCAGGGACAGGCACCATTTA-3ʹ |
| mbcl-2 forward | 5ʹ- TGACTTCTCTCGTCGCTACCGT-3ʹ |
| mbcl-2 reverse | 5ʹ- CCTG AAGAGTTCCTCCACCACC-3ʹ |
| mbax forward | 5ʹ- GCAAACTGGTGCTCAAG GCC-3ʹ |
| mbax reverse | 5ʹ- GGCCTTCCTAATGCCAACCT -3ʹ |
| mIFN-γ forward | ATGAACGCTACACACTGCATC |
| mIFN-γ reverse | CCATCCTTTTGCCAGTTCCTC |
| hIFN-γ forward | TCGGTAACTGACTTGAATGTCCA |
| hIFN-γ reverse | TCGCTTCCCTGTTTTAGCTGC |
| hIL-12b forward | TGCCCATTGAGGTCATGGTG |
| hIL-12b reverse | CTTGGGTGGGTCAGGTTTGA |
| mIL-12b forward | TGGTTTGCCATCGTTTTGCTG |
| mIL-12b reverse | ACAGGTGAGGTTCACTGTTTCT |
