# Supplementary material for: Unraveling the role of host kinase PIM1 in Toxoplasma gondii infection: Implications for therapies
Source: PLoS Negl Trop Dis. 2026 Jan 20;20(1):e0013915. doi: 10.1371/journal.pntd.0013915 (PMC12844532; doi:10.1371/journal.pntd.0013915)
Supplement: Attachment — Submitted filename: PNTD-D-24-01850_reviewer.pdf [file pntd.0013915.s007.pdf]

PLOS Neglected Tropical Diseases

Unraveling the role of host kinase PIM1 in <i>Toxoplasma gondii</i> infection: implications for therapies

--Manuscript Draft--

**Manuscript Number:**	PNTD-D-24-01850
**Full Title:**	Unraveling the role of host kinase PIM1 in <i>Toxoplasma gondii</i> infection: implications for therapies
**Short Title:**	Unraveling the role of host kinase PIM1 in <i>Toxoplasma gondii</i> infection
**Article Type:**	Research Article
**Keywords:**	<i>Toxoplasma gondii</i>; PIM1 kinase; PIM1 inhibitor; apoptosis; host-parasite interaction
**Abstract:**	<i>Toxoplasma gondii</i>, a widespread intracellular protozoan parasite, infects a significant portion of the global human population. This study elucidates the role of the host protein PIM1, a serine/threonine protein kinase, in facilitating <i>T. gondii</i> proliferation and its potential as a therapeutic target. Employing both in vitro and in vivo models, we establish that PIM1 enhances the intracellular proliferation of <i>T. gondii</i> by suppressing host cell apoptosis. Our findings underscore the necessity of PIM1's kinase activity in this process, as evidenced by the significant reduction in <i>T. gondii</i> proliferation upon treatment with either a kinase-dead PIM1 mutant or the PIM1 inhibitor AZD1208. In murine models, AZD1208 treatment resulted in decreased <i>T. gondii</i> load and elevated pro-apoptotic markers in tissues, indicating that PIM1 inhibition bolsters the host's immune response against the parasite. These results not only shed light on the intricate host-parasite interactions but also position PIM1 as a promising target for the development of anti-<i>T. gondii</i> therapeutics.
**Additional Information:**	
**Question**	**Response**
**Government Employee**<br><br>Are you or any of the contributing authors an employee of the United States government?<br><br>Manuscripts authored by one or more US Government employees are not copyrighted, but are licensed under a CC0 Public Domain Dedication, which allows unlimited distribution and reuse of the article for any lawful purpose. This is a legal requirement for US Government employees.<br><br>This will be typeset if the manuscript is accepted for publication.	No - No authors are employees of the U.S. government.
**Financial Disclosure**<br><br>Enter a financial disclosure statement that describes the sources of funding for the	Yes

work included in this submission. Review the submission guidelines for detailed requirements. View published research articles from *PLOS Neglected Tropical Diseases* for specific examples.<br><br>This statement is required for submission and **will appear in the published article** if the submission is accepted. Please make sure it is accurate.<br><br>**Funded studies**<br>Enter a statement with the following details:<br>• Initials of the authors who received each award<br>• Grant numbers awarded to each author<br>• The full name of each funder<br>• URL of each funder website<br>• Did the sponsors or funders play any role in the study design, data collection and analysis, decision to publish, or preparation of the manuscript?<br><br>Did you receive funding for this work?	
Please add funding details.<br>as follow-up to "**Financial Disclosure**<br><br>Enter a financial disclosure statement that describes the sources of funding for the work included in this submission. Review the submission guidelines for detailed requirements. View published research articles from *PLOS Neglected Tropical Diseases* for specific examples.<br><br>This statement is required for submission and **will appear in the published article** if the submission is accepted. Please make sure it is accurate.	National Natural Science Foundation (81802003); Scientific Research Foundation of Education Department of Anhui Province of China (2022AH050751); Anhui Medical University Doctoral Scientific Research Foundation (xj201725); Anhui Province New Era Talent Cultivation Quality Project (2022jyjxggyj224)

**Funded studies**<br>Enter a statement with the following details:<br>• Initials of the authors who received each award<br>• Grant numbers awarded to each author<br>• The full name of each funder<br>• URL of each funder website<br>• Did the sponsors or funders play any role in the study design, data collection and analysis, decision to publish, or preparation of the manuscript?<br><br>Did you receive funding for this work?"	
Please select the country of your main research funder (please select carefully as in some cases this is used in fee calculation).<br>as follow-up to "**Financial Disclosure**<br><br>Enter a financial disclosure statement that describes the sources of funding for the work included in this submission. Review the submission guidelines for detailed requirements. View published research articles from *PLOS Neglected Tropical Diseases* for specific examples.<br><br>This statement is required for submission and **will appear in the published article** if the submission is accepted. Please make sure it is accurate.<br><br>**Funded studies**<br>Enter a statement with the following details:<br>• Initials of the authors who received each award<br>• Grant numbers awarded to each author<br>• The full name of each funder<br>• URL of each funder website<br>• Did the sponsors or funders play any role in the study design, data collection and analysis, decision to publish, or preparation of the manuscript?<br><br>Did you receive funding for this work?"	CHINA - CN
**Competing Interests**	The authors have declared that no competing interests exist

On behalf of all authors, disclose any competing interests that could be perceived to bias this work.<br><br>This statement will be typeset if the manuscript is accepted for publication.<br><br>Review the instructions link below and PLOS NTDs' competing interests policy to determine what information must be disclosed at submission.	
**Data Availability**<br><br>Provide a **Data Availability Statement** in the box below. This statement should detail where the data used in this submission can be accessed. This statement will be typeset if the manuscript is accepted for publication.<br><br>Before publication, authors are required to make all data underlying their findings fully available, without restriction. Review our PLOS Data Policy page for detailed information on this policy. Instructions for writing your Data Availability statement can be accessed via the Instructions link below.	All relevant data are within the manuscript and its Supporting Information files

Manuscript Click here to access/download;Manuscript;manuscript.pdf

Unraveling the role of host kinase PIM1 in *Toxoplasma gondii* infection: implications for therapies

Lijie Pan[1¶], Fan Zhang[1¶], Yue Sun[1,3¶], Lingling Song[1], Famin Zhang[1], Jinjin Zhu[1], Yang Wang[1], Chong Wang[1], Qingli Luo[1], Wen Zhang[2*], Li Yu[1*], Yuanyuan Cao[1*]

1 Department of Microbiology and Parasitology, Anhui Province Laboratory of Zoonoses, School of Basic Medical Sciences, Anhui Medical University, Hefei, Anhui, China, 2 Hefei First People's Hospital, the Third Affiliated Hospital of Anhui Medical University, 3 Clinical College of Anhui Medical University.

¶These authors contributed equally to this work.

* Corresponding author: E-mail: vickycao1989@126.com (YYC); lilyyu33@126.com (LY); zw112200@126.com (WZ).

Abstract:

Toxoplasma gondii, a widespread intracellular protozoan parasite, infects a significant portion of the global human population. This study elucidates the role of the host protein PIM1, a serine/threonine protein kinase, in facilitating *T. gondii* proliferation and its potential as a therapeutic target. Employing both in vitro and in vivo models, we establish that PIM1 enhances the intracellular proliferation of *T. gondii* by suppressing host cell apoptosis. Our findings underscore the necessity of PIM1's kinase activity in this process, as evidenced by the significant reduction in *T. gondii* proliferation upon treatment with either a kinase-dead PIM1 mutant or the PIM1 inhibitor AZD1208. In murine models, AZD1208 treatment resulted in decreased *T. gondii* load and elevated pro-apoptotic markers in tissues, indicating that PIM1 inhibition bolsters the host's immune response against the parasite. These results not only shed light on the intricate host-parasite interactions but also position PIM1 as a promising target for the

development of anti-*T. gondii* therapeutics.

Keywords: *Toxoplasma gondii*, PIM1 kinase, PIM1 inhibitor, apoptosis, host-parasite interaction.

Author Summary

PIM1 functions as a regulator that suppresses apoptosis and, consequently, fosters the proliferation of *T. gondii*. This promotion of *T. gondii* proliferation by PIM1 is contingent upon its kinase activity, and the inhibition of PIM1 has been shown to induce increased apoptosis in host cells across both in vitro and in vivo studies. The potential therapeutic application of AZD1208, a PIM1 small molecule inhibitor, in treating toxoplasmosis has been brought to light by these findings. AZD1208 has demonstrated the ability to eliminate *T. gondii*-infected cells and decrease the overall parasite load by inhibiting PIM1. Collectively, the research emphasizes the role of PIM1 in the proliferation of *T. gondii* during infection and highlights the therapeutic potential of PIM1 inhibition in the fight against toxoplasmosis. The study's findings propose that targeting PIM1 may offer a viable strategy for the development of innovative treatments aimed at this intracellular parasite.

Introduction

Toxoplasma gondii, a zoonotic intracellular protozoan parasite, is renowned for its ability to invade and infect virtually all nucleated cells within its host. The transmission of *T. gondii* to humans predominantly occurs through the consumption of undercooked meat harboring viable tissue cysts or raw meat, as well as through the ingestion of food and water contaminated with *T. gondii* oocysts. Globally, nearly all warm-blooded

vertebrates, including humans, act as intermediate hosts, with cats and other members of the Felidae family serving as definitive hosts (1, 2). A systematic review and prevalence snapshot investigation revealed that the global mean seroprevalence rate of *T. gondii* infection stands at 25.7% (3). The genus *Toxoplasma* includes only one species, *Toxoplasma gondii*. *T. gondii* is classified into type I strains (such as RH, GT1, etc.), type II strains (such as PRU, ME49, etc.), and type III strains (such as VEG, etc.). Different genotypes of *T. gondii* possess distinct genetic backgrounds, which significantly influence their pathogenicity to hosts and their ability to form cysts within the host. Type I strains exhibit the highest virulence, while Type II and III strains show relatively lower pathogenicity. Despite their lower pathogenicity, Type II and III strains are more prone to cyst formation within the host and may possess a higher cyst-forming capacity compared to Type I strains (4-6). Currently, the therapeutic regimen for toxoplasmosis primarily involves the combination of pyrimethamine and sulfadiazine(7). Despite the established efficacy of this regimen, it is recognized that these treatments fall short in completely eradicating tissue cysts, leading to the requirement for extended treatment durations in immunosuppressed individuals. This limitation poses significant challenges in the management of toxoplasmosis, highlighting the critical need for the research and development of novel anti-*T. gondii* chemotherapeutic agents and vaccines.

Infection by *T. gondii* prompts the host's immune system to utilize a variety of pattern recognition receptors (PRRs) to detect pathogen-associated molecular patterns (PAMPs) specific to *T. gondii*(8-13). This recognition process initiates the production of

inflammatory cytokines and chemokines, which are essential for the host's defense mechanisms. Notably, the interleukin (IL)-12/interferon (IFN)-γ axis is particularly crucial in orchestrating the host's response to *T. gondii* infection(14, 15). Moreover, apoptosis serves as a critical host mechanism for eliminating intracellular pathogens. Initiation of apoptosis, particularly through the intrinsic pathway, involves the activation and oligomerization of pro-apoptotic Bcl-2 family proteins, such as Bax and Bak. This process leads to an increased permeability of the mitochondrial outer membrane, facilitating the release of cytochrome c into the cytosol. The cytosolic cytochrome c then promotes the formation of the apoptosome, which activates caspase-9. Subsequently, caspase-9 triggers the activation of downstream effector caspases, including caspase-3. This cascade of events culminates in nuclear condensation, DNA fragmentation, alterations in cell membrane composition, and the formation of apoptotic bodies, ultimately resulting in cell death(16, 17). Extensive research has established that *T. gondii* can suppress host cell apoptosis, targeting both the mitochondrial and death receptor pathways, which is vital for the parasite's survival and replication within host cells(18-24).

Despite extensive research describing the anti-apoptotic effects of *T. gondii* in human cells, the parasite factors that trigger this response and the host proteins that may play a role in this process remain elusive. Some studies have explored host dependency factors in *T. gondii*-infected cells, including certain amino acids, host proteins, and miRNAs (25-27). Among these, we have taken note of a study that utilized whole-genome CRISPR screening to identify human host factors required for *T. gondii*

infection, with particular interest in the proviral integration of Moloney murine leukemia virus 1 protein kinase (PIM1) (28). PIM1 is a serine/threonine protein kinase that belongs to the PIM kinase family which plays significant role in cellular proliferation, differentiation, and survival, including anti-apoptotic functions(29, 30). In addition, it is associated with the development of various types of cancer(31). Given its role in cancer, PIM1 is considered a potential target for anticancer drug development(32-34). Researchers are developing small molecule inhibitors that can suppress the activity of PIM1, such as AZD1208(35-37). However, the role of PIM1 in vivo during *T. gondii* infection and the treatment of toxoplasmosis by AZD1208 is rarely studied.

Our research has revealed that PIM1 promotes the proliferation of ME49 which is classified as Type II *T. gondii*, an effect primarily dependent on its kinase activity. Furthermore, similar results were obtained in mice, where treatment with the PIM1 inhibitor AZD1208 significantly reduced *T. gondii* load. We have confirmed both in vitro and in vivo that PIM1 promotes *T. gondii* proliferation by inhibiting apoptosis. Collectively, these findings enhance our understanding of the interactions between host cells and *T. gondii* and provide potential targets for the development of anti-*T. gondii* therapeutics.

Materials and methods

Ethics statement

The animal experiments were conducted with the approval of the Animal Experimental Ethics Committee at Anhui Medical University, under permit number 20180243,

adhering to the institution's Guidelines for the Care and Use of Research Animals. The animals were maintained in a controlled environment with regulated temperature and humidity, subjected to a 12-hour light/dark cycle, and provided with unlimited access to food and filtered water within standard enclosures.

Cell and Parasite Culture

African Green Monkey Kidney (Vero) cells, Henrietta Lacks (HeLa) cells, Human Embryonic Kidney (HEK 293T) cells, and Mouse Leukemia Monocyte Macrophage (Raw264.7) cells were cultured in Dulbecco's Modified Eagle Medium (DMEM, Viva Cell), supplemented with 10% fetal bovine serum (FBS, NEWZERUM), 100 μg/ml penicillin and 100 μg/ml streptomycin (Sigma, USA). Cultures were maintained in an incubator at 37°C with 5%CO_2. The *T. gondii* ME49 strain was cultured in Vero cells using DMEM containing 2% FBS. Lysates from *T. gondii*-infected Vero cells were purified by sequential centrifugation at 800 rpm for 5 minutes to collect the supernatant, followed by a second centrifugation at 3500 rpm for 5 minutes to clear the supernatant. The *T. gondii* tachyzoites were resuspended in DMEM with 2% FBS and enumerated before infecting the cells at a multiplicity of infection (MOI) of 3 for 24 hours.

RNA/DNA Extraction and Quantitative Real-Time PCR Analysis

Total RNA was extracted from cells and mouse spleens using Trizol Reagent (Invitrogen, USA). This RNA was reverse transcribed using the EvoM-MLV RT Kit with gDNA Clean for qPCR (AG, China), following the manufacturer's protocol. Genomic DNA was extracted from the same samples using the SteadyPure Universal Genomic DNA Extraction Kit (AG, China). cDNA synthesis was conducted using the

SYBR Green Premix Pro Taq HS qPCR Kit (AG, China) on a LightCycler 96 fluorescence quantitative PCR instrument (Roche, Switzerland). Primer sequences are hGAPDH forward, 5′- ATGACATCAAGAAGGTGGTG-3′; hGAPDH reverse, 5′-CATACCAGGAAATGAGCTTG-3′; *Tg*ITS1 forward, 5′- AATATTGGAAGCCAGT GCAGG-3′; *Tg*ITS1 reverse, 5′- CAATCTTTCACTCTCTCTCAA-3′; m-GAPDH forward, 5′- ATGGTGAAGGTCGGTGTGAA-3′ ;m-GAPDH reverse, 5′- CGCTCC TGGAAGATGGTGAT-3′; m-PIM1 forward, 5′- TACACGGACTTTGATGGG ACC-3′; m-PIM1 reverse, 5′- CTCAGGGACAGGCACCATTTA-3′; m-BCL-2 forward, 5′-TGACTTCTCTCGTCGCTACCGT-3′; m-BCL-2 reverse, 5′- CCTG AAGAGTTCCTCCACCACC-3′; m-BAX forward, 5′- GCAAACTGGTGCTCAAG GCC-3′; m-BAX reverse, 5′- GGCCTTCCTAATGCCAACCT -3′. Relative cDNA levels were determined using the 2-ΔΔCT method, with expression values normalized to the housekeeping gene GAPDH. Each sample was analyzed in triplicate and each experiment was repeated a minimum of three times.

Protein Isolation and Western Blot Analysis

Cells were washed with PBS and lysed in a solution containing cold RIPA Lysis buffer (Beyotime, China), 1% Phenylmethanesulfonyl fluoride (PMSF, Beyotime), and Protease Inhibitor Cocktail (MCE, USA). Spleen tissues were homogenized in the same lysis buffer, and proteins were extracted. Protein samples were mixed with 5× SDS loading buffer and boiled for 5 minutes. Lysates were resolved on a 15% SDS-PAGE gel and transferred to a PVDF membrane (Billerica, USA). Membranes were blocked with 5% nonfat skim milk in TBST (0.1% Tween 20) for 1.5 hours at room temperature, then incubated with primary antibodies overnight at 4°C, followed by incubation with horseradish peroxidase (HRP)-conjugated secondary antibodies for 1 hour at room temperature. Protein bands were visualized using the ChemiDoc Chemiluminescence Platform (Bio-Rad, USA) and Sparkjade ECL Super (Sparkjade, Shandong, China).

Primary antibodies were used at the following dilutions: anti-β-Actin (Proteintech, China) 1:3000, Bcl-2 (Proteintech, China) 1:2000, Cleaved-Caspase-3 (Proteintech, China) 1:2000, and Bax (Proteintech, China) 1:2000.

Giemsa Staining

Cells were cultured on glass coverslips in 12-well plates and infected with *T. gondii* ME49 for 24 hours. Following incubation, the medium was discarded, and the coverslips were washed three times with cold PBS, fixed with 4% paraformaldehyde for 30 minutes, and then washed again with cold PBS. The cells were stained with Giemsa staining solution (Beyotime, China) for 30 minutes and gently rinsed. After air-drying, the coverslips were mounted onto slides using neutral resin for microscopic examination.

TUNEL Apoptosis Staining

T. gondii ME49-infected cells were subjected to the TUNEL Apoptosis Detection Kit (Beyotime, China), with a final step of counterstaining with Antifade Mounting Medium with DAPI (Beyotime, China). The cells were subsequently observed under a fluorescence microscope.

Animal Model

Female C57BL/6J mice, aged five weeks, were intraperitoneally injected with 1×10^5 *T. gondii* ME49 strain one day prior to the experiment. The mice were randomly assigned to two groups and treated with either DMSO or AZD1208(MCE, USA) at a dosage of 30 mg/kg/dose for seven consecutive days. Spleens and livers were harvested on day seven. All procedures adhered to the internationally accepted guidelines for the

ethical treatment of laboratory animals.

Results

PIM1 promotes intracellular *T. gondii* proliferation

In our preliminary research, we aimed to ascertain the regulatory function of PIM1 kinase as a putative host factor in modulating the proliferation of type II ME49 strains of *T. gondii*. To achieve this, we utilized a combination of quantitative real-time polymerase chain reaction (qRT-PCR) and western blot (WB) analyses to meticulously examine the impact of PIM1 on the proliferation patterns of this specific *T. gondii* strain across a panel of genetically engineered cell lines. Specifically, we compared the parasite burden of *T. gondii* the in PIM1-null HeLa cells, Raw264.7 cells with stable PIM1 knockdown, and 293T cells with transient PIM1 transfection, juxtaposed against their corresponding wild-type and control cell lines. Our findings revealed that in the absence or reduced expression of PIM1, there was a significant downregulation of the mRNA levels of the *T. gondii*-specific gene internal transcribed spacer-1 (*Tg*ITS-1) (Figure 1A and 1B). Conversely, overexpression of PIM1 was associated with an increase in *Tg*ITS-1 mRNA levels (Figure 1C). Additionally, we scrutinized the expression of the *T. gondii* profilin protein. Suppression of PIM1 expression resulted in a diminished profilin protein level (Figure 1D and 1E), while overexpression of PIM1 also led to an elevation in profilin protein levels (Figure 1F). The validation of PIM1 expression levels in the genetically modified cell lines was confirmed through WB (Figure 1G-I).

Upon invasion of host cells, *T. gondii* encases itself within a specialized membrane

structure known as the parasitophorous vacuole membrane (PVM). Utilizing Giemsa staining, we quantified the number of *T. gondii* organisms within PVs in both PIM1-null HeLa cells and their wild-type counterparts. Upon PIM1 knockout, the number of tachyzoites per PV reduced remarkablely compared to that in wild-type cells (Figure 1J). For ease of observation, we have conducted a quantitative analysis of the number of *T. gondii* within the PVs. (Figure 1K). These results showed that the intracellular proliferation of *T. gondii* is compromised in the absence of PIM1. These findings provide compelling evidence that both exogenous and endogenous PIM1 kinases significantly enhance the intracellular proliferation of *T. gondii*. These data indicate that the intracellular proliferation of *T. gondii* is compromised in the absence of PIM1, suggesting that PIM1 plays a pivotal role in facilitating the parasite's life cycle within the host cell.

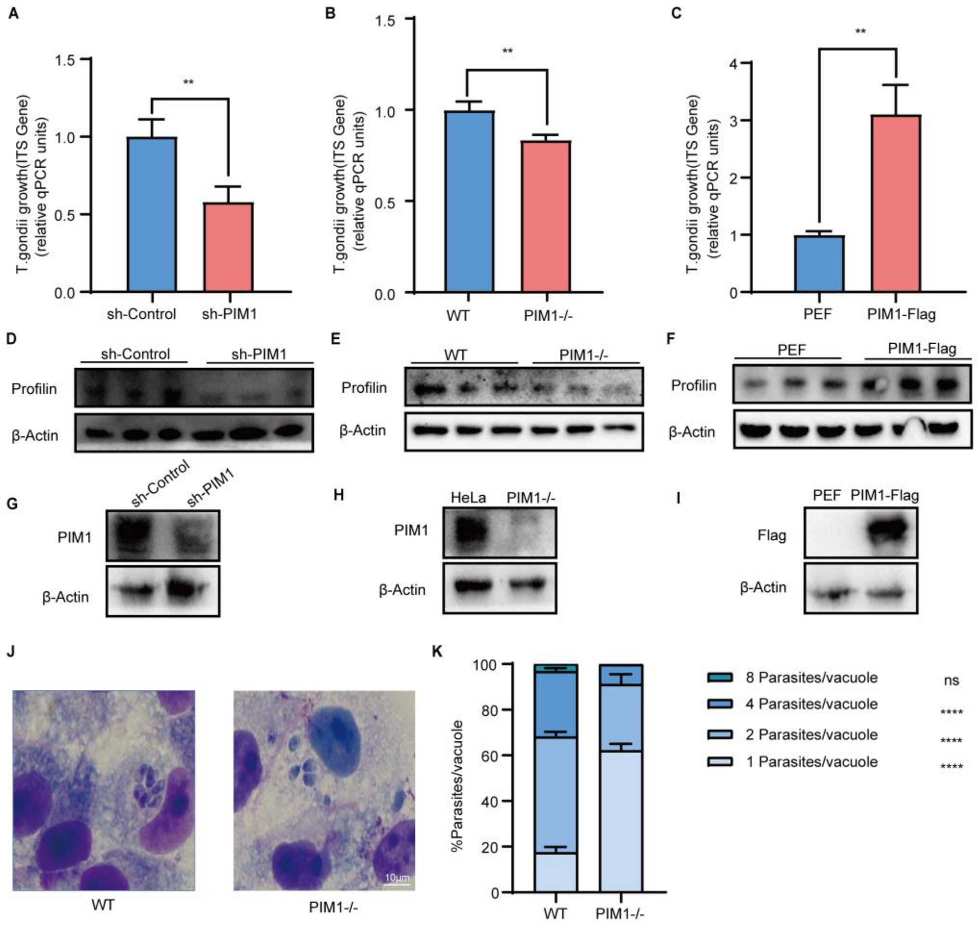

Figure 1. PIM1 enhances the intracellular burden of *T. gondii* in infected cells.

(A-C) qRT-PCR analysis was performed to assess the expression levels of *Tg*ITS-1 in the PIM1-deficient HeLa cells, PIM1-depleted RAW264.7 cells, HEK293T cells transfected with PIM1-Flag and the corresponding control group subsequent to *T. gondii* infection. (D-F) *T. gondii* proliferation was analyzed by anti-Profilin and β-actin immunoblotting. (G-I) WB detection of endogenous or exogenous PIM1 protein expression in HeLa cells, RAW264.7 cells and HEK293T cells. (J) Giemsa staining was performed to visualize *T. gondii* within PVs in wild-type and PIM1 knockout HeLa cells after infection. (K) Quantitative bar chart analysis of the parasite load within PVs, derived from Giemsa staining data. Data presented are the averages from three independent experiments and were analyzed using a two-tailed unpaired t-test. Graphs

display the mean ± s.d. (n=3) from three independent experiments. ns for not significant, Data presented are the averages from three independent experiments and were analyzed using a two-tailed unpaired t-test. Graphs display the mean ± s.d. (n=3) from three independent experiments. ns for not significant, ***P < 0.001, ****P < 0.0001.

PIM1 Promotes *T. gondii* proliferation in kinase activity-Dependent Manner

To ascertain the necessity of PIM1's kinase activity for its role in facilitating *T. gondii* proliferation, we employed a kinase-dead mutant of PIM1, specifically the PIM1-K67R variant. Our experimental findings indicate that 293T cells transfected with this mutant were incapable of enhancing the levels of *Tg*ITS-1 mRNA, in contrast to cells overexpressing wild-type PIM1, which significantly amplified the expression of *Tg*ITS-1 mRNA when compared to those transfected with an empty vector (Figure 2A). Furthermore, the application of the PIM1 kinase inhibitor AZD1208 resulted in a pronounced suppression of *Tg*ITS-1 mRNA levels relative to the control group (Figure 2B). Additionally, the profilin protein levels were examined, revealing that overexpression of PIM1 substantially increased the profilin protein levels, an effect not observed with the PIM1-K67R mutant (Figure 2C). Consistent with these observations, the inhibitory impact of AZD1208 on PIM1 kinase activity abrogated the promotion of proliferation (Figure 2D), thereby underscoring the indispensable nature of PIM1's kinase activity for its functional role in the context of *T. gondii* intracellular

development. These results collectively highlight the pivotal role of PIM1's kinase activity in the intracellular life cycle of *T. gondii* and its potential as a therapeutic target

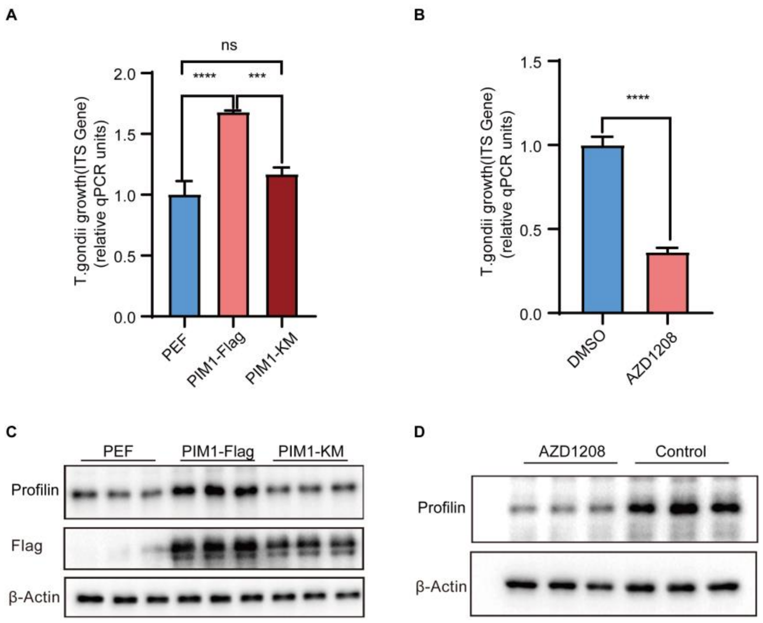

for interventions against toxoplasmosis.

Figure 2. PIM1 enhances the replication of *T. gondii* through a kinase activity-dependent mechanism. (A and B) qRT-PCR assays were conducted to ascertain the relative expression levels of the *Tg*ITS-1, in HEK293T cells following transient transfection with the designated plasmids, as well as in both DMSO-treated control and AZD1208-treated RAW264.7 cells. (C and D) The replication of *T. gondii* was evaluated by immunoblotting with anti-Profilin and anti-β-actin antibodies. The efficiency of PIM1 transfection was determined by immunoblotting with an anti-Flag antibody. Data presented are the averages from three independent experiments and were analyzed using a two-tailed unpaired t-test. Graphs display the mean ± s.d. (n=3) from three independent experiments. ns for not significant, ***$P < 0.001$, ****$P < 0.0001$.

PIM1 promotes the proliferation of *T. gondii* by inhibiting the level of apoptosis in the host cell

In our preceding investigations, we discerned that PIM1 is instrumental in augmenting the proliferation of *T. gondii*. Subsequently, we endeavored to delineate the underlying mechanisms mediating its effects. The apoptosis of host cells infected by *T. gondii* is acknowledged to be a pivotal factor in bolstering the humoral immune response against the parasites. Thus, we scrutinized the modulations in cellular apoptosis levels during *T. gondii* infection under conditions of PIM1 downregulation. Our experimental observations in Raw264.7 cells, in which PIM1 was stably knocked down (Figure 3C), revealed a significant downregulation of the mRNA levels of the anti-apoptotic gene bcl-2 and a concomitant upregulation of the mRNA levels of the pro-apoptotic gene bax, in comparison to the control group (Figure 3A and 3B). Additionally, we quantified the protein levels of Bcl-2, Bax, and the apoptotic marker cleaved-Caspase3. It was observed that post-*T. gondii* infection, the downregulation of PIM1 corresponded with a diminution in Bcl-2 protein levels, concurrent with an elevation in Bax and cleaved-Caspase3 protein levels (Figure 3D and 3E). These observations suggest that the suppression of PIM1 expression subsequent to *T. gondii* infection is correlated with an upregulation of pro-apoptotic gene expression at both the transcriptional and translational levels, alongside a downregulation of anti-apoptotic gene expression within the cellular environment.

The Terminal deoxynucleotidyl transferase dUTP Nick End Labeling (TUNEL)

staining is a highly sensitive technique designed to detect DNA double-strand breaks that are exposed during the apoptosis process by recognizing the 3'-termini. This method is capable of identifying even minute quantities of cellular apoptosis. Employing TUNEL staining on Raw264.7 cells with stable PIM1 knockdown and the control group, *T. gondii* infection or not, we assessed cellular apoptosis. Our findings indicated that in the uninfected group, the suppression of PIM1 was associated with an enhanced incidence of apoptosis. Moreover, upon *T. gondii* infection, a decrease in PIM1 protein expression was correlated with a further escalation in cellular apoptosis levels (Figure 3F-I). The TUNEL staining substantiated that following *T. gondii* infection, PIM1 contributes to the proliferation of the parasite by inhibiting the onset of cellular apoptosis. In aggregate, these data imply that PIM1 exerts an inhibitory influence on apoptosis, potentially playing a critical role in promoting the intracellular survival and proliferation of *T. gondii*.

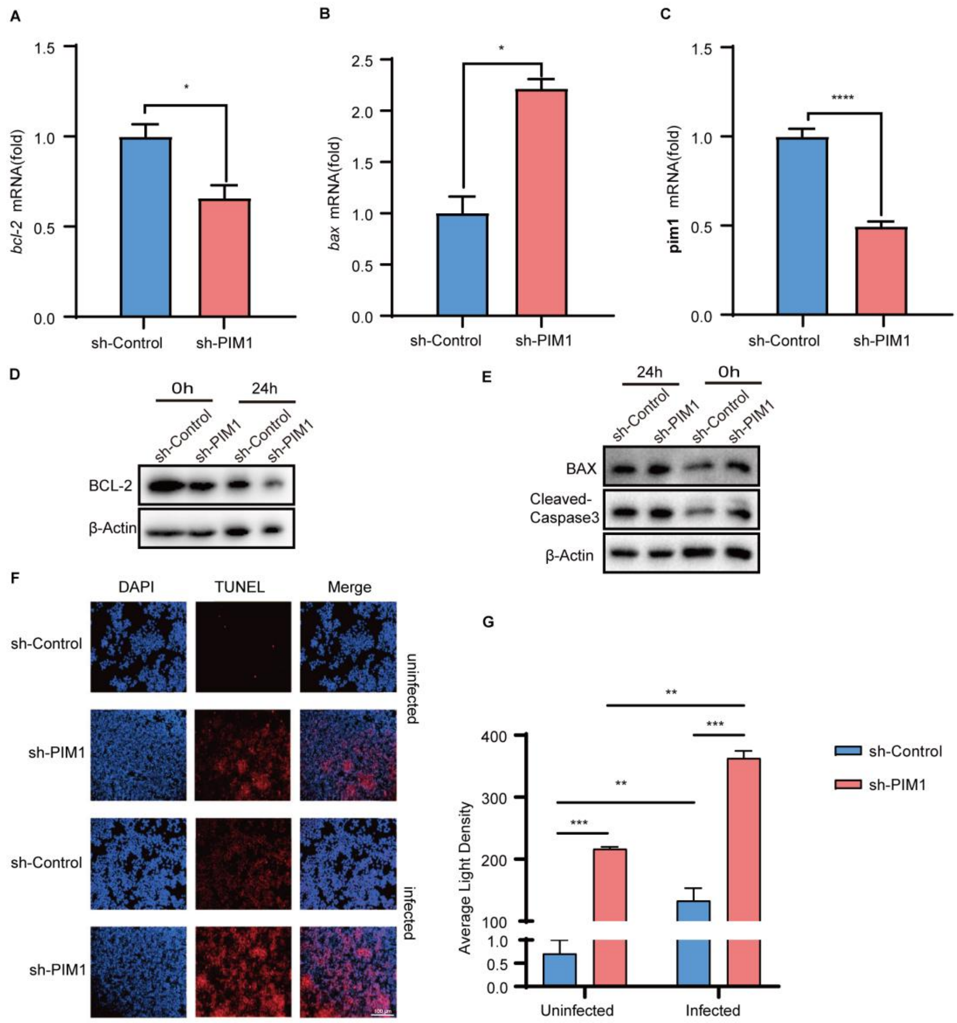

Figure 3. PIM1 facilitates the induction of apoptosis in cells infected with *T.gondii.*

(A-C) qRT-PCR analysis was conducted to quantify the mRNA expression levels of bcl-2, bax, and pim1 in both control and PIM1 stably knockdown RAW264.7 cells following *T. gondii* infection. (D-E) WB assays were employed to assess the protein abundance of BCL-2, BAX, and Cleaved-Caspase3 in the control group as well as in PIM1 stably knockdown RAW264.7 cells post-infection. (F) Apoptosis was visualized using TUNEL staining in both uninfected and *T. gondii*-infected control cells, as well as in PIM1 stably knockdown RAW264.7 cells. (I) A quantitative analysis of the

fluorescence images in F. Data presented are the averages from three independent experiments and were analyzed using a two-tailed unpaired t-test. Graphs display the mean ± s.d. (n=3) from three independent experiments. *P < 0.05, **P < 0.01, ***P < 0.001, ****P < 0.0001.

AZD1208 inhibits the proliferation of *T. gondii* in vivo

In prior in vitro cellular assays, evidence has accrued to suggest that the serine/threonine kinase PIM1 is instrumental in the proliferation of *T. gondii*. The present study endeavors to elucidate whether this regulatory role extends to the in vivo context within murine models. Building upon the established mechanistic insights into PIM1's facilitation of *T. gondii* replication, we are poised to ascertain the congruence of this function in a physiological milieu. Leveraging the documented safety and efficacy profile of AZD1208 as an anti-neoplastic agent in mice, this investigation extends its application to the realm of infectious disease research.

The experimental paradigm commenced with the inoculation of mice with ME49 tachyzoites of *T. gondii*, followed by a regimen of daily intraperitoneal injections of AZD1208 for a period of seven days, with the objective of attenuating PIM1 kinase activity. Splenic and hepatic tissues were excised for subsequent analysis of *T. gondii* burden and quantification of cytokine profiles indicative of anti-parasitic immune responses (Figure 4A). The data revealed a marked diminution in the tissue load of *T. gondii* within the spleen and liver of mice subjected to PIM1 inhibition, in stark contrast to the untreated control cohort (Figure 4B and 4E). Furthermore, the interrogation of cytokine expression post-inhibitor intervention disclosed a pronounced upregulation in

the hepatic and splenic mRNA levels of IFN-γ (Figure 4C and 4F) and IL-12 (Figure 4D and 4G), cytokines pivotal to the immunological cascade against *T. gondii*. Collectively, these observations underscore the pivotal role of PIM1 in *T. gondii* proliferation in vivo.

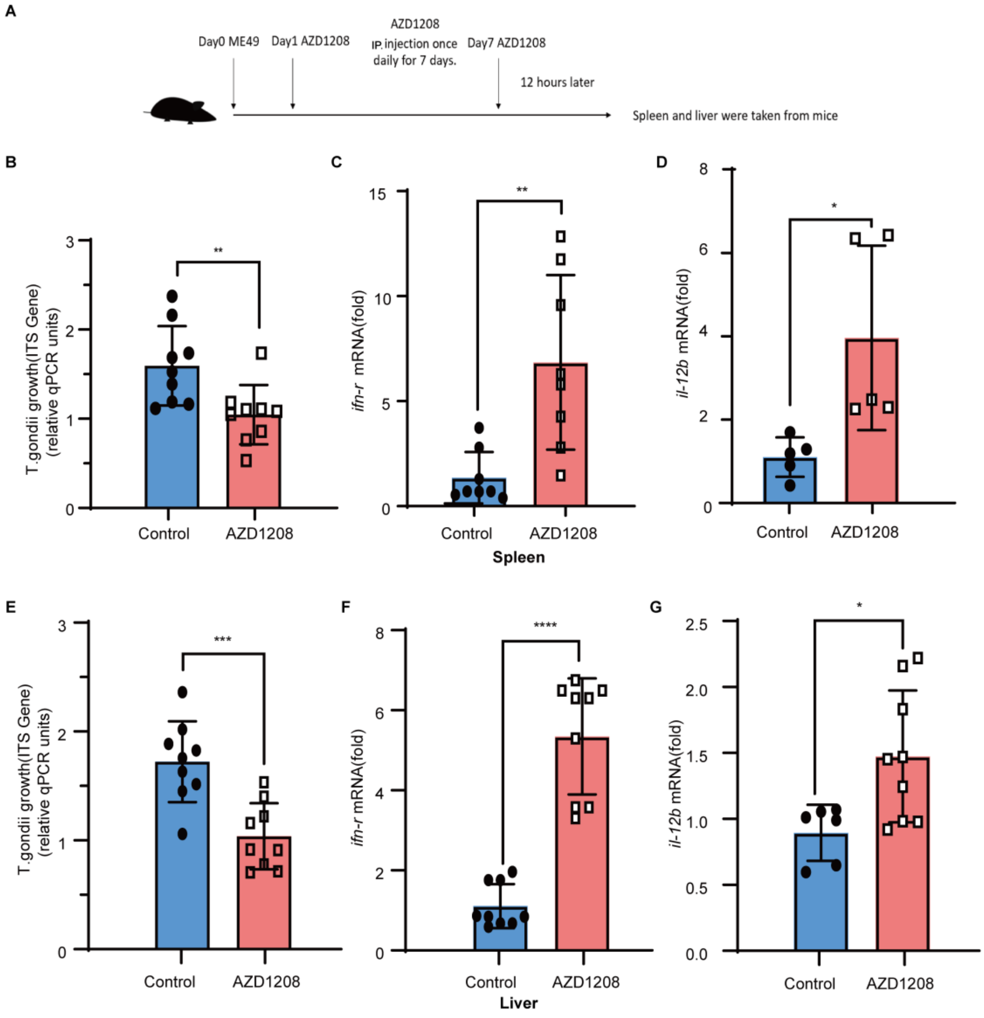

Figure 4. PIM1 Inhibitor Diminishes Parasite Burden in Mice Infected with *T. gondii*. (A) An overview of the in vivo infection model is depicted. (B-D) qRT-PCR analysis was conducted to assess the mRNA expression levels of the *Tg*ITS-1, as well as IFN-γ and IL-12, in spleen tissues from mice treated with AZD1208 versus those left untreated following *T. gondii* infection. (E-G) qRT-PCR was utilized to evaluate the

mRNA expression levels of *Tg*ITS-1, IFN-γ, and IL-12 in liver tissues from AZD1208-treated and untreated mice post-infection. Data presented are the averages from three independent experiments and were analyzed using a two-tailed unpaired t-test. Graphs display the mean ± s.d. (n=3) from three independent experiments. *P < 0.05, **P < 0.01, ***P < 0.001, ****P < 0.0001.

AZD1208 induced apoptosis in vivo

Subsequent to the delineation of PIM1's role in the murine model, the study proceeded to dissect the underpinning mechanisms by which PIM1 may be orchestrating *T. gondii* proliferation. Drawing from cellular studies, PIM1 has been implicated in facilitating *T. gondii* replication by suppressing apoptosis, we hypothesized a conserved mechanism in the in vivo. To this end, RNA and protein extracts from the splenic tissues of the aforementioned murine model were subjected to qRT-PCR and WB analysis. The experimental data demonstrated a significant downregulation of bcl-2 mRNA and a corresponding upregulation of bax mRNA within the splenic tissues of mice administered with AZD1208 subsequent to *T. gondii* infection, as compared to the untreated control group (Figure 5A and 5B). In concert with these transcriptional changes, the WB analysis revealed a notable reduction in Bcl-2 protein levels alongside a significant elevation in Bax protein levels and the presence of the cleaved form of Caspase3, indicative of heightened apoptotic activity in AZD1208-treated mice post-*T. gondii* infection (Figure 5C-5E). Next to it is the bar chart of the quantitative analysis of the WB bands. These results collectively indicate that the suppression of PIM1 activity subsequent to *T. gondii* infection is correlated with an augmentation of

apoptotic markers within murine splenic tissue, which implicates PIM1 as a pivotal suppressor of apoptosis that promotes the replication of *T. gondii*. The modulation of PIM1 activity may thus represent a critical cellular mechanism that influences the host-pathogen interplay during *T. gondii* infection. The convergence of these in vivo and in vitro observations has led to the compelling conclusion that PIM1 exerts its pro-replicative effects on *T. gondii*, in part, through the suppression of apoptotic pathways.

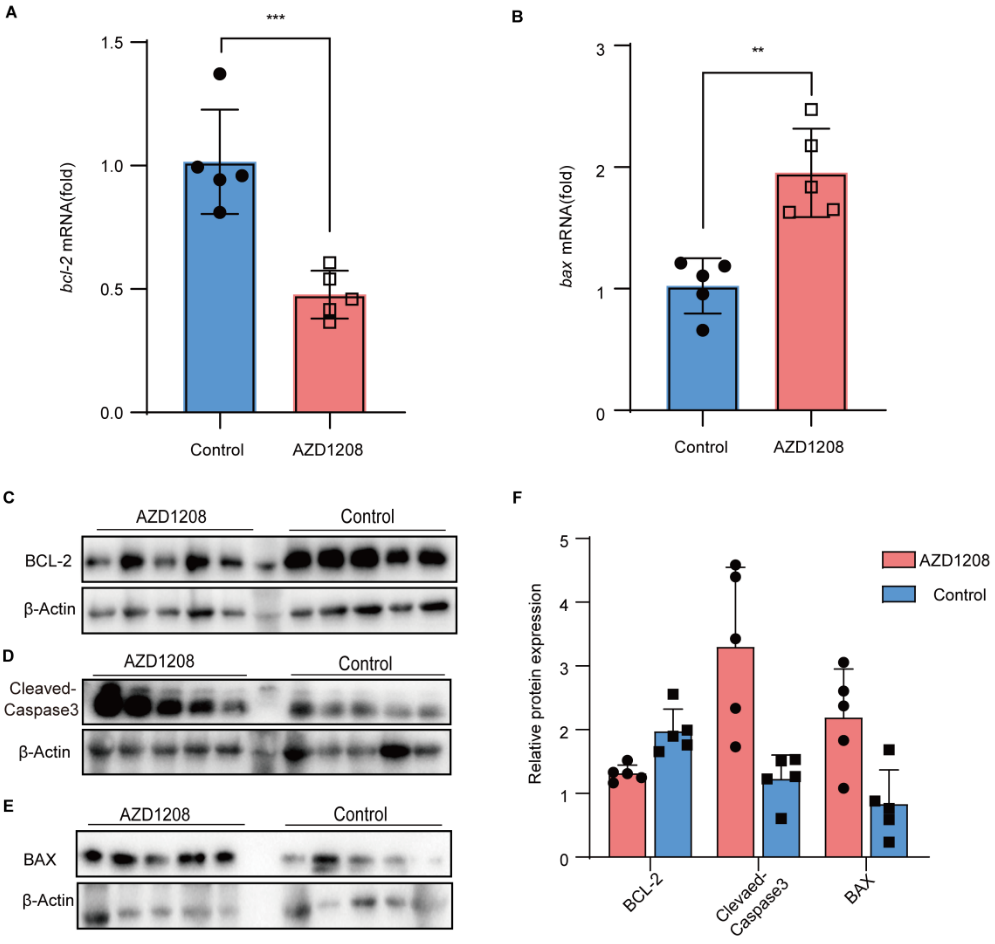

Figure 5. In Vivo Apoptosis Induction by PIM1 Inhibitor Treatment in Mice Infected with *T. gondii*. (A-B) qRT-PCR was employed to assess the mRNA expression levels of the bcl-2 and bax in spleen from mice treated with the PIM1 inhibitor

AZD1208 compared to those in the untreated cohort following *T. gondii* infection. (C-E) WB analysis was conducted to determine the protein levels of the apoptosis markers BCL-2, cleaved-caspase3, and BAX in splenic tissues of AZD1208-treated and untreated mice post-infection. (F) A quantitative analysis of the WB band intensities in C-E. Data presented are the averages from three independent experiments and were analyzed using a two-tailed unpaired t-test. Graphs display the mean ± s.d. (n=3) from three independent experiments. **P < 0.01, ***P < 0.001.

Discussion

The development of therapeutic strategies against *T. gondii* has been challenging due to its intracellular lifestyle and its ability to evade the host immune response. The complexity of *T. gondii*'s interaction with its host is further highlighted by the parasite's ability to manipulate host cell machinery to its advantage. However, the identification of PIM1 as a key host factor in *T. gondii* proliferation offers a promising avenue for the development of targeted therapies.

Our findings, corroborated by the extensive body of research on *T. gondii*, underscore the significance of PIM1 in the intracellular survival and proliferation of the parasite within its host cells. The role of PIM1 in promoting *T. gondii* proliferation, as demonstrated in both in vitro and in vivo models, positions it as a potential therapeutic target for toxoplasmosis. The inhibition of PIM1 activity, achieved through the use of the kinase inhibitor AZD1208, resulted in a significant reduction of *T. gondii* load in murine models, highlighting the potential of PIM1 inhibitors as a novel treatment strategy. The modulation of host cell apoptosis by *T. gondii* is a critical aspect of the

parasite's survival strategy. Our results, which show that PIM1 suppresses apoptosis and thereby promotes *T. gondii* proliferation, are consistent with the notion that the parasite manipulates host cell death pathways to its advantage.

Guanylate binding protein 1 (GBP1), a GTPase, is induced by IFN-γ and plays a crucial role in the immune response against pathogens (38, 39). Activated during the inflammatory process, GBP1 has the potential to attack cellular membranes as well as plasma membrane derived vacuoles such as PVM of *T. gondii* and disrupt their integrity (40). In the context of *T. gondii* infection, GBP1 is capable of targeting the parasites within the infected cells(41, 42). PIM1 is known to regulate GBP1 activity, which is crucial for preventing self-harm and defending against infections. It was reported that during *T. gondii* infection, the virulence protein *Tg*IST disrupts IFN-γ signaling and depletes PIM1, increasing GBP1 activity. PIM1 plays the role in maintaining the integrity of IFN-γ signaling and as a potential bait for pathogen virulence factors(43). Furthermore, in our study, PIM1 was verified that it promoted *T. gondii* proliferation through inhibiting apoptosis while the specific *T. gondii* protein that interacts with PIM1 to suppress apoptosis remains to be elucidated, and our forthcoming studies are aimed at identifying this key protein and elucidate this interaction, which is crucial for understanding the molecular mechanisms by which *T. gondii* manipulates PIM1. The manipulation of host cell apoptosis by *T. gondii* is a testament to its adaptability and the sophisticated strategies it employs to ensure its survival within the host.

In conclusion, our research highlights the importance of PIM1 in the complex host-parasite relationship with *T. gondii*. The inhibition of PIM1 activity presents a

promising avenue for the development of targeted therapies against toxoplasmosis, with potential implications for the treatment of other diseases influenced by this protozoan parasite (Figure 6).

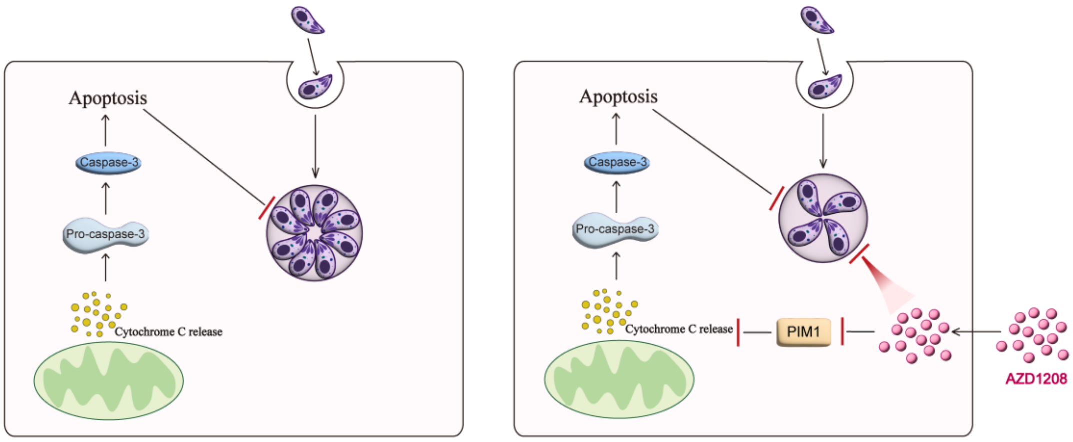

Figure 6. GRAPH ABSTRACT: The character of PIM1 in regulating proliferation of *T. gondii*. PIM1 facilitated the proliferation of *T. gondii* via suppressing apoptosis. AZD1208, a small molecule inhibitor of PIM1, led to the elimination of *T. gondii* and consequently reduced the parasite load.

References

1. Montoya JG, Liesenfeld O. Toxoplasmosis. Lancet. 2004;363(9425):1965-76.
2. Milne G, Webster JP, Walker M. Toxoplasma gondii: AnUnderestimated Threat? Trends Parasitol. 2020;36(12):959-69.
3. Molan A, Nosaka K, Hunter M, Wang W. Global status of Toxoplasma gondii infection: systematic review and prevalence snapshots. Trop Biomed. 2019;36(4):898-925.
4. Darde ML, Bouteille B, Pestre-Alexandre M. Isoenzyme analysis of 35 Toxoplasma gondii isolates and the biological and epidemiological implications. J Parasitol. 1992;78(5):786-94.
5. Sibley LD, Boothroyd JC. Virulent strains of Toxoplasma gondii comprise a single clonal lineage. Nature. 1992;359(6390):82-5.
6. Howe DK, Sibley LD. Toxoplasma gondii comprises three clonal lineages: correlation of parasite genotype with human disease. J Infect Dis. 1995;172(6):1561-6.
7. Aerts R, Mehra V, Groll AH, Martino R, Lagrou K, Robin C, et al. Guidelines for the management of Toxoplasma gondii infection and disease in patients with haematological malignancies and after haematopoietic stem-cell transplantation: guidelines from the 9th European Conference on Infections in Leukaemia, 2022. Lancet Infect Dis. 2024;24(5):e291-e306.
8. Golding H, Aliberti J, King LR, Manischewitz J, Andersen J, Valenzuela J, et al. Inhibition of HIV-1 infection by a CCR5-binding cyclophilin from Toxoplasma gondii. Blood. 2003;102(9):3280-

6.
9. Yarovinsky F, Zhang D, Andersen JF, Bannenberg GL, Serhan CN, Hayden MS, et al. TLR11 activation of dendritic cells by a protozoan profilin-like protein. Science. 2005;308(5728):1626-9.
10. Plattner F, Yarovinsky F, Romero S, Didry D, Carlier MF, Sher A, Soldati-Favre D. Toxoplasma profilin is essential for host cell invasion and TLR11-dependent induction of an interleukin-12 response. Cell Host Microbe. 2008;3(2):77-87.
11. Ewald SE, Chavarria-Smith J, Boothroyd JC. NLRP1 is an inflammasome sensor for Toxoplasma gondii. Infect Immun. 2014;82(1):460-8.
12. Hu Z, Wu D, Lu J, Zhang Y, Yu SM, Xie Y, et al. Inflammasome Activation Dampens Type I IFN Signaling to Strengthen Anti-Toxoplasma Immunity. mBio. 2022;13(6):e0236122.
13. Jin QW, Yu T, Pan M, Fan YM, Ge CC, He XB, et al. Toxoplasma gondii ROP5 Enhances Type I IFN Responses by Promoting Ubiquitination of STING. Int J Mol Sci. 2024;25(20).
14. Suzuki Y, Orellana MA, Schreiber RD, Remington JS. Interferon-gamma: the major mediator of resistance against Toxoplasma gondii. Science. 1988;240(4851):516-8.
15. Suzuki Y, Conley FK, Remington JS. Importance of endogenous IFN-gamma for prevention of toxoplasmic encephalitis in mice. J Immunol. 1989;143(6):2045-50.
16. Lima TS, Lodoen MB. Mechanisms of Human Innate Immune Evasion by Toxoplasma gondii. Front Cell Infect Microbiol. 2019;9:103.
17. Bertheloot D, Latz E, Franklin BS. Necroptosis, pyroptosis and apoptosis: an intricate game of cell death. Cell Mol Immunol. 2021;18(5):1106-21.
18. Nash PB, Purner MB, Leon RP, Clarke P, Duke RC, Curiel TJ. Toxoplasma gondii-infected cells are resistant to multiple inducers of apoptosis. J Immunol. 1998;160(4):1824-30.
19. Goebel S, Luder CG, Gross U. Invasion by Toxoplasma gondii protects human-derived HL-60 cells from actinomycin D-induced apoptosis. Med Microbiol Immunol. 1999;187(4):221-6.
20. Annis MG, Soucie EL, Dlugosz PJ, Cruz-Aguado JA, Penn LZ, Leber B, Andrews DW. Bax forms multispanning monomers that oligomerize to permeabilize membranes during apoptosis. EMBO J. 2005;24(12):2096-103.
21. Carmen JC, Hardi L, Sinai AP. Toxoplasma gondii inhibits ultraviolet light-induced apoptosis through multiple interactions with the mitochondrion-dependent programmed cell death pathway. Cell Microbiol. 2006;8(2):301-15.
22. Vutova P, Wirth M, Hippe D, Gross U, Schulze-Osthoff K, Schmitz I, Luder CG. Toxoplasma gondii inhibits Fas/CD95-triggered cell death by inducing aberrant processing and degradation of caspase 8. Cell Microbiol. 2007;9(6):1556-70.
23. Hippe D, Weber A, Zhou L, Chang DC, Hacker G, Luder CG. Toxoplasma gondii infection confers resistance against BimS-induced apoptosis by preventing the activation and mitochondrial targeting of pro-apoptotic Bax. J Cell Sci. 2009;122(Pt 19):3511-21.
24. Friedrich A, Pechstein J, Berens C, Luhrmann A. Modulation of host cell apoptotic pathways by intracellular pathogens. Curr Opin Microbiol. 2017;35:88-99.
25. Gaji RY, Huynh MH, Carruthers VB. A novel high throughput invasion screen identifies host actin regulators required for efficient cell entry by Toxoplasma gondii. PLoS One. 2013;8(5):e64693.
26. Moser LA, Pollard AM, Knoll LJ. A genome-wide siRNA screen to identify host factors necessary for growth of the parasite Toxoplasma gondii. PLoS One. 2013;8(6):e68129.
27. Menendez MT, Teygong C, Wade K, Florimond C, Blader IJ. siRNA Screening Identifies the Host Hexokinase 2 (HK2) Gene as an Important Hypoxia-Inducible Transcription Factor 1 (HIF-1)

Target Gene in Toxoplasma gondii-Infected Cells. mBio. 2015;6(3):e00462.
28. Wu SZ, Wei HX, Jiang D, Li SM, Zou WH, Peng HJ. Genome-Wide CRISPR Screen Identifies Host Factors Required by Toxoplasma gondii Infection. Front Cell Infect Microbiol. 2019;9:460.
29. Wang Z, Bhattacharya N, Weaver M, Petersen K, Meyer M, Gapter L, Magnuson NS. Pim-1: a serine/threonine kinase with a role in cell survival, proliferation, differentiation and tumorigenesis. J Vet Sci. 2001;2(3):167-79.
30. Macdonald A, Campbell DG, Toth R, McLauchlan H, Hastie CJ, Arthur JSC. Pim kinases phosphorylate multiple sites on Bad and promote 14-3-3 binding and dissociation from Bcl-X. Bmc Cell Biol. 2006;7.
31. Narlik-Grassow M, Blanco-Aparicio C, Carnero A. The PIM family of serine/threonine kinases in cancer. Med Res Rev. 2014;34(1):136-59.
32. Shah N, Pang B, Yeoh KG, Thorn S, Chen CS, Lilly MB, Salto-Tellez M. Potential roles for the PIM1 kinase in human cancer - A molecular and therapeutic appraisal. Eur J Cancer. 2008;44(15):2144-51.
33. Ogawa N, Yuki H, Tanaka A. Insights from Pim1 structure for anti-cancer drug design. Expert Opin Drug Discov. 2012;7(12):1177-92.
34. Bellon M, Nicot C. Targeting Pim kinases in hematological cancers: molecular and clinical review. Mol Cancer. 2023;22(1).
35. Keeton EK, McEachern K, Dillman KS, Palakurthi S, Cao YC, Grondine MR, et al. AZD1208, a potent and selective pan-Pim kinase inhibitor, demonstrates efficacy in preclinical models of acute myeloid leukemia. Blood. 2014;123(6):905-13.
36. Kirschner AN, Wang J, van der Meer R, Anderson PD, Franco-Coronel OE, Kushner MH, et al. PIM Kinase Inhibitor AZD1208 for Treatment of MYC-Driven Prostate Cancer. Jnci-J Natl Cancer I. 2015;107(2).
37. Cortes J, Tamura K, DeAngelo DJ, de Bono J, Lorente D, Minden M, et al. Phase I studies of AZD1208, a proviral integration Moloney virus kinase inhibitor in solid and haematological cancers. Brit J Cancer. 2018;118(11):1425-33.
38. Kim BH, Shenoy AR, Kumar P, Das R, Tiwari S, MacMicking JD. A family of IFN-gamma-inducible 65-kD GTPases protects against bacterial infection. Science. 2011;332(6030):717-21.
39. Fisch D, Bando H, Clough B, Hornung V, Yamamoto M, Shenoy AR, Frickel EM. Human GBP1 is a microbe-specific gatekeeper of macrophage apoptosis and pyroptosis. EMBO J. 2019;38(13):e100926.
40. Bass AR, Egan MS, Alexander-Floyd J, Lopes Fischer N, Doerner J, Shin S. Human GBP1 facilitates the rupture of the Legionella-containing vacuole and inflammasome activation. mBio. 2023;14(5):e0170723.
41. Qin A, Lai DH, Liu Q, Huang W, Wu YP, Chen X, et al. Guanylate-binding protein 1 (GBP1) contributes to the immunity of human mesenchymal stromal cells against Toxoplasma gondii. Proc Natl Acad Sci U S A. 2017;114(6):1365-70.
42. Fisch D, Clough B, Domart MC, Encheva V, Bando H, Snijders AP, et al. Human GBP1 Differentially Targets Salmonella and Toxoplasma to License Recognition of Microbial Ligands and Caspase-Mediated Death. Cell Rep. 2020;32(6):108008.
43. Fisch D, Pfleiderer MM, Anastasakou E, Mackie GM, Wendt F, Liu X, et al. PIM1 controls GBP1 activity to limit self-damage and to guard against pathogen infection. Science. 2023;382(6666):eadg2253.
