# Supplementary material for: Unraveling the role of host kinase PIM1 in Toxoplasma gondii infection: Implications for therapies
Source: PLoS Negl Trop Dis. 2026 Jan 20;20(1):e0013915. doi: 10.1371/journal.pntd.0013915 (PMC12844532; doi:10.1371/journal.pntd.0013915)
Supplement: Attachment — Submitted filename: Response to Reviewers.docx [file pntd.0013915.s009.docx]

Dear Dr. McCall,

I am pleased to submit our revised manuscript to PLOS Neglected Tropical Diseases. We have carefully considered the feedback from the editor and reviewers and have made the necessary revisions to address their concerns. With those improvements, we hope that the current version could meet the standards of of PLOS Neglected Tropical Diseases.

There is one point that needs to be clarified with you: we have added a new author, Yun Yang, to the manuscript. This is due to the significant contributions she made during the major revision process.

I sincerely appreciate all the support you have provided during the revision of the manuscript. Below is my point-by-point response to the reviewers' comments.

Best regards,

Yuanyuan Cao

Point-by-point responses to the comments of reviewers:

The reviewer’s comments are in Arial font, which are followed by our responses in underlined Times New Roman font.

**Reviewers' Comments:**

Reviewer's Responses to Questions

**Key Review Criteria Required for Acceptance?**


As you describe the new analyses required for acceptance, please consider the following:


**Methods**


-Are the objectives of the study clearly articulated with a clear testable hypothesis stated?

-Is the study design appropriate to address the stated objectives?

-Is the population clearly described and appropriate for the hypothesis being tested?

-Is the sample size sufficient to ensure adequate power to address the hypothesis being tested?

-Were correct statistical analysis used to support conclusions?

-Are there concerns about ethical or regulatory requirements being met?

Reviewer #1: See below comments

Reviewer #2: While the study objectives are generally well-defined, it would be beneficial to explicitly state the hypothesis in terms of specific molecular mechanisms by which PIM1 influences *T. gondii* proliferation. The study design, combining in vitro and in vivo approaches, is appropriate, but the lack of clarity regarding the duration of mouse infection compromises reproducibility. The cell populations and animal model are described, but justifying the choice of the ME49 strain and discussing the generalization of the results to other strains would be important. The sample size (n=3) suggests adequate power, but a brief justification based on statistical power analysis would be helpful. The statistical tests (t-test) seem appropriate, but more details and justifications are needed. Finally, a more detailed statement regarding compliance with ethical guidelines for animal research is essential.

Answer: We sincerely appreciate your meticulous review of our study and your valuable comments. We have carefully considered and improved upon the issues you raised. We have stated the hypothesis in the lines 125-138 and also carefully elaborated on the duration of mouse infection in lines 239-252 of the Methods section. We have explained the rationale for choosing the ME49 strain in lines 115 and lines 263-265 and discussed the possibility of generalizing the results to other strains in the lines 283-288 of the Results section and lines 521-526 of the Discussion section. Regarding the statistical tests (t-tests), we have provided more details and justifications in the figure legends or in the Methods section (lines 253-259). Finally, we have elaborated on compliance with the ethical guidelines for animal research in the lines 140-155 in Methods section.

Reviewer #3: The objectives of the study is articulated well and is timely with a clear hypothesis. However, the study design could be more robust, for instances, authors should use type I and type III strains of Toxoplasma to show that the effect of PIM1 is not specific to host response against a specific Toxoplasma strain. Also, authors should also test the *Tg*IST1 KO parasites as it can activate the host IFN-responses much robustly compared to the Wild-type parasites.Statistical tests were done correctly and ethical requirements were met.

Answer: Thank you very much for your constructive suggestions. In addition to the type II ME49 strain, we have already examined the effects of PIM1 on the proliferation of the classic type I RH strain and the Chinese-specific *T. gondii* Chinese 1 genotype Wh6 strain (abbreviated as *Tg*Ctwh6). As shown in Supplementary Figure 1, deficiency of PIM1 expression resulted in a diminished profilin protein level, indicating that PIM1 can also promote the proliferation of the RH strain and the Chinese-specific *Tg*Ctwh6 strain. This finding supports the universality of PIM1's effect across different strains. Regarding *Tg*IST1 knockout parasites, your suggestion is highly valuable. Since our research group does not currently have the relevant parasite strains, we will consider including *Tg*IST1 knockout parasites in future studies to further explore their impact on the host IFN response.

**Results**


-Does the analysis presented match the analysis plan?

-Are the results clearly and completely presented?

-Are the figures (Tables, Images) of sufficient quality for clarity?

Reviewer #1: See below comments

Reviewer #2: In general, the analysis presented corresponds to the plan outlined in the methodology. The authors use qRT-PCR, Western blot, Giemsa staining, and TUNEL staining to assess *T. gondii* proliferation and cellular apoptosis, as described. However, the lack of details about the statistical tests used makes it difficult to fully assess the appropriateness of the analysis.
The results are presented generally clearly, with figures and tables that illustrate the main findings. However, some additional information would be helpful to increase clarity:
Indicate the internal control genes used in the qRT-PCR graphs.
Mention the concentration of the AZD1208 inhibitor used and the justification for this choice.
Provide information about the number of cells analyzed in each experiment (e.g., Giemsa staining, TUNEL staining)
The figures are of good quality and easy to interpret.
Suggestions:In the qRT-PCR results (Figures 1A, 1B, 1C), indicate which genes were used as internal controls (e.g., GAPDH).
In Figure 1J, increase the resolution of the images for better visualization of the parasites.

Answer: We sincerely appreciate your suggestions. GAPDH served as the endogenous control in all qRT-PCR analyses, as indicated in respective figure legends. We have also specified the concentration of the AZD1208 inhibitor in the figure legend (lines 355) and the Methods section (lines 239-252). Specifically, the concentration of AZD1208 used in cells is 5 µM, and in animals, it is 30 mg/kg. The rationale for this choice is based on the manufacturer's instructions and relevant literature, and we have provided an explanation in the corresponding results section (lines 332-334). We have provided the number of cells analyzed in each experiment in the Methods section. In Figure 1J, we have replaced the image with a higher resolution one and annotated the *T. gondii* with red arrows.

Reviewer #3: The analysis and the interpretation of the data needs a lot of improvement. Firstly, authors need a consistent data throughout. For example, authors wrote in HeLa cells, they use Sh-PIM1 in Fig. 1A but then in Fig. 1J they used PIM1 KO HeLa cells! which one is true KO or knockdown using Sh RNA?
Secondly, Western blot pictures for Cleaved Caspase 3 is not convincing at all and same for all other markers. Authors should show uninfected, WT and uninfected knockdown cells for all the markers as well as the total Caspase 3.
For all the western blot picture, full unprocessed blots should be given in supplementary materials.
Thirdly, Tunnel assay pictures are not at all convincing. Authors should show with higher magnification and also to show that the parasites are infected in the cells using SAG1/GRA7 staining in parallel for which commercially available antibodies are there.
In Figure 5, the loading of different wells are extremely unequal and is of poor quality.
Finally, throughout most of the experiments, critical controls are missing. How do we know the effect of the drugs in the uninfected mice also!! and also, authors should use a positive control drug which will negate the effects.

Answer: We sincerely appreciate your comments. We have carefully elaborated on the differences between the two cell types in the Results section. Specifically, in Figures 1A, D and G, we used hairpin RNA to knock down the expression of the Pim1 gene in Raw264.7 cells. In Figures 1B, E, H and J, we utilized CRISPR-Cas9 technology to knock out the expression of the Pim1 gene in HeLa cells. In Figures 1C, F and I, we overexpressed PIM1 using transient transfection technology. In the manuscript, there were some places where the expression was not clear, leading to confusion. We have now revised these sections to enhance clarity and ensure that our intentions are clearly conveyed.

Regarding your second question, we have added detection of total Caspase 3 and controls for uninfected wild-type and uninfected knockdown cells, as shown in Figure 3F. We have also provided the complete unprocessed Western blot images in the supplementary materials. For your third question, we have used higher magnification and demonstrated the parasitic infection in the cells using antibodies against *T. gondii* GAP45 in Figure 3D (Green Fluorescence). For Figure 5, we have repeated the experiment to ensure equal loading. In the mice model, we have added controls for uninfected mice and used the positive drug Pyrimethamine as control.

**Conclusions**


-Are the conclusions supported by the data presented?

-Are the limitations of analysis clearly described?

-Do the authors discuss how these data can be helpful to advance our understanding of the topic under study?

-Is public health relevance addressed?

Reviewer #1: See below comments

Reviewer #2: The authors mention some limitations, such as the need to identify the *T. gondii* protein that interacts with PIM1. However, other important limitations are not addressed:
The generalization of the results to other strains of *T. gondii*.
The lack of investigation of the detailed molecular mechanisms by which PIM1 regulates apoptosis.
The absence of data on the efficacy of the AZD1208 inhibitor in chronic infection models.
The conclusions about the role of PIM1 in *T. gondii* proliferation and modulation of apoptosis are generally supported by the data presented, both in vitro and in vivo. However, it is important to note that the conclusions are primarily based on a single strain of *T. gondii* (ME49), it should be mentioned.
The authors discuss how the identification of PIM1 as a therapeutic target may open new avenues for the development of treatments against toxoplasmosis. They also mention the relevance of PIM1 in modulating the host immune response. However, they could further explore the translational potential of these findings, considering the development of combination therapies or the use of PIM1 inhibitors in conjunction with other drugs.
The authors mention the high prevalence of toxoplasmosis and the impact on immunocompromised individuals. However, they could further emphasize the relevance to public health by discussing:
The economic impact of toxoplasmosis (treatment costs, loss of productivity).
The importance of preventing toxoplasmosis in pregnant women.
The need to develop new therapies to overcome resistance to current treatments.
The conclusions are generally supported by the data, but generalization is limited by the use of a single *T. gondii* strain. The authors mention some limitations, but do not address the generalization of the results, the detailed molecular mechanisms, and the efficacy in chronic infection models. The discussion about advancing understanding of the topic is good, but the translational potential could be further explored. The relevance to public health is mentioned, but the economic impact, prevention in pregnant women, and the need for new therapies could be emphasized.

Answer: We thank the reviewer for the insightful comments. In Supplementary Figure 1 we used WB to extend the analysis to the type I RH strain and the Chinese 1 genotype Wh6 strain. PIM1 deletion enhanced replication of both strains, just as the effect observed with the ME49 (type II) strain, indicating functional conservation of PIM1 across genotypes. In the revised Discussion (lines 521-526), we explicitly state that our study mainly focused on the ME49 strain and further explore the limitations of the study as well as the generalizability of the results.

Regarding the lack of detailed molecular insights into how PIM1 modulates apoptosis, we have now incorporated Figure 3G to demonstrate that PIM1 deficiency enhances mitochondrial cytochrome c release and cleaved caspase-3 levels after infection, indicating that PIM1 suppresses host intrinsic apoptosis (mitochondrial apoptosis pathway) to facilitate parasite replication.

Regarding AZD1208 in chronic infection: we sincerely appreciate your feedback, which is very constructive. However, the pathways of chronic infection differ from those of acute infection, and the clearance of *T. gondii* primarily occurs during the acute phase of infection. Since chronic toxoplasmosis is characterized by tissue cyst formation, its immune clearance mechanism is significantly different from the acute phase, and current clinical interventions mainly focus on controlling the acute phase. Therefore, this study focuses on the acute model. We look forward to verifying the effects of the inhibitor AZD1208 in chronic *T. gondii* infection in subsequent experiments. We agree on its importance.

In response to your suggestions, we have emphasized in the Introduction section (lines 40-62) the harmful effects of toxoplasmosis (including impacts on immunocompromised individuals and pregnant women) as well as the significant burden on the economy and society. We have also highlighted the shortcomings of current treatments and the necessity for the development of new drugs.

In the Discussion, we acknowledge the limitations of the parasite strains used in this study. However, based on the results of our in vitro experiments (Figure 1S), we believe that the potential for generalizing these findings to other strains is substantial. Additionally, we highlight in the Discussion (lines 529-538) the significant potential of PIM1 inhibitors as combination therapies, particularly for immunocompromised patients, including those with cancer.

Reviewer #3: Although the data is interesting, but currently many data is questionable which needs clear clarification and controls

Answer: We sincerely appreciate your comments. In accordance with the reviewers’ suggestions, we have refined the manuscript’s interpretations and added essential controls—including uninfected groups and positive-drug controls—to enhance the overall quality of the study.

**Editorial and Data Presentation Modifications?**


Use this section for editorial suggestions as well as relatively minor modifications of existing data that would enhance clarity. If the only modifications needed are minor and/or editorial, you may wish to recommend “Minor Revision” or “Accept”.

Reviewer #1: (No Response)

Reviewer #2: Yes, I recommend the following editorial and data presentation modifications:

Revise the writing of the introduction to make it more concise and focused, avoiding excessively generic information about *T. gondii*.
Indicate which genes were used as internal controls in the qRT-PCR results (Figures 1A, 1B, 1C).
Mention the concentration used of the AZD1208 inhibitor and the justification for this choice in the results.
Improve the resolution of the microscopy images (e.g., Figure 1J) for better visualization of the parasites."
Recommendation:

Given the other shortcomings we identified (lack of clarity about the duration of mouse infection, need for more support in the discussion), "Major Revisions" is the most appropriate recommendation.

Answer: Thank you for your valuable suggestions. We have addressed each point as follows:

The Introduction has been streamlined by removing redundant background and sharpening the focus on the study hypothesis.

All qRT-PCR figures now include a note specifying GAPDH as the internal control gene and that data were analyzed using the 2^-ΔΔCt^ method.

As mentioned above, the concentration of AZD1208 and the rationale for its selection have been added to the revised manuscripts.

Microscopy images in Figure 1J were re-acquired with a 63×oil-immersion objective at 300 dpi resolution; parasites are now clearly visible and marked with red arrows.

Reviewer #3: NA

**Summary and General Comments**


Use this section to provide overall comments, discuss strengths/weaknesses of the study, novelty, significance, general execution and scholarship. You may also include additional comments for the author, including concerns about dual publication, research ethics, or publication ethics. If requesting major revision, please articulate the new experiments that are needed.

Reviewer #1: This manuscript by Pan L and colleagues reports host protein, Moloney murine leukemia virus 1 protein kinase (PIM1), its role in pathogenesis of *Toxoplasma gondii,* infection and its potential as therapeutic target for treatment of *T. gondii* infections. They found that PIM1 inhibited apoptosis of *T. gondii*-infected host cells, which lead to enhancing intracellular proliferation of *T. gondii*. They further showed in a mouse model that the PIM1 inhibitor AZD1208 treated mice harbored lighter parasitic load upon *T. gondii* infection than the non-treated controls. They enthusiastically concluded that PIM1 is a promising target for the development of anti-*T. gondii* therapeutics.
The most important concern of this reviewer is that targeting the host protein PIM1 as therapeutics. For cancer chemotherapies, this may be considered, but not for *T. gondii* infections. PIM1 inhibitors such as AZD1208 very likely cause apoptosis of not only *T. gondii* infected host cells but also normal non-infected host cells. The line of in vitro evidence strongly suggests that AZD1208-treated mice of non-*T. gondii* infection would suffer from consequence of apoptosis, counteracting its usefulness as therapeutic agents. The authors must redirect their attention to the roles of PIM1 on the molecular pathogenesis of *T. gondii*.
In mouse model experiments, one important control is missing, which is non-*T. gondii* infected animals with AZD1208 treatment. This would show whether such a treatment is specifically to only *T. gondii*-infected host cells. This is pertinent even PIM1 is remotely considered as a therapeutic target.
An additional aspect the authors need to pay attention to is figure legends. Figure legends should be able to stand alone by themselves for readers to understand the figures without reference to main text. These include brief descriptions of methods, clear labels and what each label is, which are lack in the legends of this manuscript. What are the lanes of Western blots of the figures? There are multiple lanes with no explanation what they are.
Some methods are not presented, such as quantification of tachyzoites, protein extraction. Please see the PDF file for more information.

Answer：We sincerely appreciate your constructive feedback.Thank you very much for your insightful comments. First, we fully agree with your viewpoint. Given that post-infection most individuals remain asymptomatic, while immunocompromised patients (e.g., those with cancer) may develop severe toxoplasmosis, we have emphasized in the revised manuscript that PIM1 is a critical target for combination therapy and explicitly noted the limitations of AZD1208 (see lines 534-538). Regarding the molecular mechanism, we increased the resolution of TUNEL staining and measured cytochrome C levels in both the cytosol and mitochondria, suggesting that PIM1 may act via the intrinsic apoptotic pathway (Figure 3G). In the mouse experiments, we added uninfected controls and a positive-drug control group, as shown in Figure 4 and Figure 5. Concerning the figure legends, we have revised them to stand alone, with all changes clearly marked. The Methods section has also been amended to include previously missing details.

Reviewer #2: The manuscript addresses a relevant topic within the scope of the journal, investigating the role of host kinase PIM1 in *Toxoplasma gondii,* infection. Identifying host factors that influence parasite proliferation can open new avenues for developing therapies. Overall, the study is well-conducted and presents interesting results. However, some areas need revision to increase the clarity and impact of the work.
Strengths:
Relevance: The study focuses on a neglected tropical disease of great global importance.
Originality: The investigation of the role of PIM1 in *T. gondii* infection is an original contribution.
Methodology: The study employs a combination of in vitro and in vivo approaches.
Results: The results are clear and well-presented, with robust quantitative data.
Weaknesses:
Introduction: Could be more concise and focused, avoiding excessively generic information about *T. gondii*.
Move the information about the different strains of *T. gondii* (lines 40-47) to a later paragraph, where the relevance to the study can be better established.
In line 53, specify what the "critical need" for new therapies is (resistance, side effects, etc.).
The last sentence of the introduction (lines 95-97) is a bit vague. Better specify which "potential targets" were identified.
The introduction (lines 40-47) presents a simplified view of the genetic diversity of *T. gondii* by mentioning only the three classic lineages (Type I, II, and III). Although this classification has been historically relevant, it does not reflect the complexity of the parasite's population structure, which includes a variety of other genotypes and lineages with different virulence characteristics and geographic distribution. It is recommended to expand this section to include a more comprehensive discussion about the genetic diversity of *T. gondii*, citing recent studies that have investigated the parasite's population structure in different regions of the world (e.g., Ajzenberg et al., 2004; Lehmann et al., 2006; Su et al., 2012). In addition, it is suggested to mention the importance of genetic diversity in the parasite's adaptation to different hosts and environments, as well as in the clinical manifestations of toxoplasmosis.

Discussion: Some statements need more support in the existing literature.
Writing: There is room for improvement in the fluency and clarity of the text.

the following additional analyses and clarifications are required:

Hypothesis: Explicitly state the study's hypothesis in terms of specific molecular mechanisms by which PIM1 influences *T. gondii* proliferation (e.g., detailing the impact on apoptosis pathways).

Study Design: Correct the lack of clarity regarding the duration of mouse infection with *T. gondii* ME49 prior to the start of treatment with DMSO or AZD1208. This information is crucial for reproducibility. Indicate which genes were used as internal controls in the qRT-PCR results (Figures 1A, 1B, 1C).
Mention the concentration used of the AZD1208 inhibitor and the justification for this choice in the results.
Improve the resolution of the microscopy images (e.g., Figure 1J) for better visualization of the parasites."

Recommendation:

Given the other shortcomings we identified (lack of clarity about the duration of mouse infection, need for more support in the discussion),

Population: Justify the choice of the ME49 strain (Type II) of *T. gondii* as the model for this study. Discuss whether the results obtained with this strain can be generalized to other strains of *T. gondii*, considering potential differences in virulence and host interactions.

Sample Size: Include a brief justification for the sample size (n=3) used in each experiment. Ideally, this should include a reference to a statistical power analysis performed to ensure adequate power to detect meaningful differences.

Statistical Analysis: Provide more specific details regarding the statistical tests used. Specify whether t-tests were paired or unpaired, and whether assumptions of normality were tested. Justify the choice of statistical tests based on the characteristics of the data.

Ethics: Include a more detailed statement regarding compliance with relevant ethical and regulatory guidelines for animal research. This should include the specific guidelines followed and any measures taken to minimize animal suffering.


Indicate which genes were used as internal controls in the qRT-PCR results (Figures 1A, 1B, 1C).
Mention the concentration used of the AZD1208 inhibitor and the justification for this choice in the results.
Improve the resolution of the microscopy images (e.g., Figure 1J) for better visualization of the parasites.

Answer：Thank you for your thorough review and constructive comments. We have addressed each point as follows: The Introduction has been streamlined to avoid generic information on *T. gondii*; details on strain diversity have been moved to a paragraph that better links them to the study’s aims.

Lines 58-62 now explicitly state that the urgent need for new therapies stems from drug resistance and adverse effects.

Lines 71-89 have expanded the discussion of *T. gondii* genetic diversity, citing Ajzenberg 2004, Lehmann 2006, and Su et al. 2012, highlighting how genotype differences affect virulence and clinical outcomes.

The entire manuscript has been copy-edited for fluency and clarity.

The study hypothesis is now stated in the last paragraph of Introduction (lines 125-138): PIM1 promotes *T. gondii* proliferation by suppressing the host mitochondrial apoptosis pathway (cytochrome c/caspase-3 axis).

We have specifically elaborated on the grouping, treatment, and infection time of mice in the Methods section. GAPDH is specified as the internal control in the legends of all qRT-PCR experiments. We have specified this in all figure legends.

AZD1208 concentration and rationale are provided in revised manuscripts as mentioned above.

Figure 1J has been replaced with a 63× oil-immersion, 300-dpi high-resolution image; parasites are indicated by red arrows.

We have explained in the manuscript the rationale for selecting the ME49 strain (type II) of *T. gondii* as the model for this study and have extended our analysis to the type I RH strain and the Chinese 1 genotype Wh6 strain in cell-based experiments. Moreover, we have revised the manuscript according to your suggestions, including modifications to the statistical analysis and the ethics section.

Reviewer #3: The manuscript is interesting but the data is not having good controls and inconclusive representation. Hence, I recommend major revision for the article.

Answer：Thank you very much for your comments. We have revised the manuscript extensively in accordance with the reviewer's suggestions.

PLOS authors have the option to publish the peer review history of their article ([what does this mean?](https://track.editorialmanager.com/CL0/https://journals.plos.org/plosntds/s/editorial-and-peer-review-process%23loc-peer-review-history/1/010f01967b155469-1708bd75-1106-4ce8-8964-addc35a66bed-000000/3Zjw9P_U8HRGNL8PerQZGKtLIwHUkGekpevI7KbWyME=208)). If published, this will include your full peer review and any attached files.


If you choose “no”, your identity will remain anonymous but your review may still be made public.


**Do you want your identity to be public for this peer review?** For information about this choice, including consent withdrawal, please see our [Privacy Policy](https://track.editorialmanager.com/CL0/https://www.plos.org/privacy-policy/1/010f01967b155469-1708bd75-1106-4ce8-8964-addc35a66bed-000000/Ot_sWEsv7l5X3C61gXD8Yvs1BC4SMCCf8hTQhukKrSw=208).

Reviewer #1: No

Reviewer #2: No

Reviewer #3: No
