# Supplementary material for: Unraveling the role of host kinase PIM1 in Toxoplasma gondii infection: Implications for therapies
Source: PLoS Negl Trop Dis. 2026 Jan 20;20(1):e0013915. doi: 10.1371/journal.pntd.0013915 (PMC12844532; doi:10.1371/journal.pntd.0013915)
Supplement: Attachment — Submitted filename: response to reviewers point by point.docx [file pntd.0013915.s010.docx]

Dear editors,

I am delighted to resubmit our revised manuscript entitled “Unraveling the role of host kinase PIM1 in Toxoplasma gondii infection: implications for therapies” for consideration at PLOS Neglected Tropical Diseases. We thank you and the reviewers for the constructive comments that have markedly strengthened the study. All concerns have been carefully addressed, as detailed in the accompanying point-by-point response.

We appreciate your continued support and hope that the revised version is now acceptable for publication in PLOS Neglected Tropical Diseases.

Best regards,

Yuanyuan Cao

On behalf of all co-authors

PNTD-D-24-01850R1

Unraveling the role of host kinase PIM1 in Toxoplasma gondii infection: implications for therapies

PLOS Neglected Tropical Diseases

Dear Dr. Cao,

Thank you for submitting your manuscript to PLOS Neglected Tropical Diseases. After careful consideration, we feel that it has merit but does not fully meet PLOS Neglected Tropical Diseases's publication criteria as it currently stands. Therefore, we invite you to submit a revised version of the manuscript that addresses the points raised during the review process.

Please submit your revised manuscript within 60 days Nov 08 2025 11:59PM. If you will need more time than this to complete your revisions, please reply to this message or contact the journal office at plosntds@plos.org. When you're ready to submit your revision, log on to https://www.editorialmanager.com/pntd/ and select the 'Submissions Needing Revision' folder to locate your manuscript file.

Please include the following items when submitting your revised manuscript:

* A rebuttal letter that responds to each point raised by the editor and reviewer(s). You should upload this letter as a separate file labeled 'Response to Reviewers'. This file does not need to include responses to any formatting updates and technical items listed in the 'Journal Requirements' section below.

* A marked-up copy of your manuscript that highlights changes made to the original version. You should upload this as a separate file labeled 'Revised Manuscript with Track Changes'.

* An unmarked version of your revised paper without tracked changes. You should upload this as a separate file labeled 'Manuscript'.

If you would like to make changes to your financial disclosure, competing interests statement, or data availability statement, please make these updates within the submission form at the time of resubmission. Guidelines for resubmitting your figure files are available below the reviewer comments at the end of this letter.

We look forward to receiving your revised manuscript.

Kind regards,

Laura-Isobel McCall

Section Editor

PLOS Neglected Tropical Diseases

Laura-Isobel McCall

Section Editor

PLOS Neglected Tropical Diseases

Shaden Kamhawi

co-Editor-in-Chief

PLOS Neglected Tropical Diseases

orcid.org/0000-0003-4304-636XX

Paul Brindley

co-Editor-in-Chief

PLOS Neglected Tropical Diseases

orcid.org/0000-0003-1765-0002

Additional Editor Comments :

Overall, reviewers appreciated your efforts in revising the paper. However, some issues remain from reviewers 2 and 3 that must be addressed. Reviewer 3 particularly noted concerns with regards to PIM1 expression levels being higher than expected. Authors should provide details on how protein identity in Western blots was confirmed, since many of the raw Western blot files provided lack molecular weight markers.

Journal Requirements:

1) Please ensure that the Title in your manuscript file is written correctly.

Note: Please correct the spelling of this word "implicatof ions" as the title in the manuscript is currently as follows: "Unraveling the role host kinase PIM1 in Toxoplasma gondii infection: implicatof ions for therapies."

Response: We sincerely appreciate your meticulous review of our study and your valuable comments. We have corrected the typo "implicatof ions" to "implications" in the title.

2) Please amend your detailed Financial Disclosure statement. This is published with the article. It must therefore be completed in full sentences and contain the exact wording you wish to be published.

1) State what role the funders took in the study. If the funders had no role in your study, please state: "The funders had no role in study design, data collection and analysis, decision to publish, or preparation of the manuscript."

Response: Thank you very much for your guidance. We have now amended our detailed Financial Disclosure statement to include the sentence: "The funders had no role in study design, data collection and analysis, decision to publish, or preparation of the manuscript."

Note: If the reviewer comments include a recommendation to cite specific previously published works, please review and evaluate these publications to determine whether they are relevant and should be cited. There is no requirement to cite these works unless the editor has indicated otherwise.

Reviewers' Comments:

Reviewer's Responses to Questions

Key Review Criteria Required for Acceptance?

As you describe the new analyses required for acceptance, please consider the following:

Methods

-Are the objectives of the study clearly articulated with a clear testable hypothesis stated?

-Is the study design appropriate to address the stated objectives?

-Is the population clearly described and appropriate for the hypothesis being tested?

-Is the sample size sufficient to ensure adequate power to address the hypothesis being tested?

-Were correct statistical analysis used to support conclusions?

-Are there concerns about ethical or regulatory requirements being met?

Reviewer #1: (No Response)

Reviewer #2: Please see Summary and General Comments

Reviewer #3: Statistical analysis are not correct. Following should be done -

1. For Figure 1K, Two Way ANOVA with multiple comparison tests(as it has two variables: WT vs KO cells and different number of parasites/vacuoles)

2. Figure 2A, One Way ANOVA (Three groups) with multiple comparison tests

3. Figure 3E, Two Way ANOVA with multiple comparison tests similar reasons as stated for Figure 1K

4. Figures 4B-F: All of them should be done using One Way ANOVA with multiple comparison tests

5. Figures 5A-D: All of them should be done using One Way ANOVA with multiple comparison tests

Response: Thank you very much for your constructive comments. All suggested changes have been implemented: Figure 1K: now analyzed by two-way ANOVA with multiple comparisons. Figure 2A: one-way ANOVA with multiple comparisons. Figure 3E: two-way ANOVA with multiple comparisons. Figures 4B-F & 5A-D: one-way ANOVA with multiple comparisons. The figure legends and Methods subsection "Statistical analysis" have been updated accordingly.

Results

-Does the analysis presented match the analysis plan?

-Are the results clearly and completely presented?

-Are the figures (Tables, Images) of sufficient quality for clarity?

Reviewer #1: (No Response)

Reviewer #2: Please see Summary and General Comments

Reviewer #3: In Figure 3, it is clearly appears from the representative images (both from previous and the new version) that just by depleting PIM1, cells are vulnerable to die and authors should provide the graph for % Tunnel positive cells too rather than just showing MFI! and discuss the result accordingly. Also, Still its not clear about the mechanism by which PIM1 mediates the protection of the parasites, a clear discussion is necessary.

Another surprising fact is that, PIM1 is inducible protein (by IFN and other cytokines) and the baseline expression is low, not only that, its an extremely short lived protein. I am surprised that how authors have detected such a thick band in WT cells? without any proteasome inhibitors! Authors need to clarify.

Furthermore, one of the major problem is most of the western blot picture are not having any molecular weight marker, how one can understand that the band appears at correct molecular weight size? I made this comment in the earlier version too, but authors did not answer to that point.

Response: We thank the reviewer for raising this critical point.

We have added a new bar graph showing the percentage of TUNEL-positive cells and updated the legend in Figure 3E and line 399 to 400.

We have expanded the Discussion (lines 547 to 551) to clarify that PIM1 appears to protect parasites primarily by suppressing mitochondria-mediated apoptosis in host cell. In addition, we also addressed the need for future identification of the parasite factor(s) involved in line 575-577.

We agree that PIM1 is an inducible protein. In the current study the intention was to demonstrate the consequence of PIM1 deletion/knock-down, not to quantify its physiological abundance. The Western blot shown is an over-exposed image that was intentionally prolonged to accentuate the difference between WT and KO/knock down samples; the apparent "thick" band therefore does not reflect high basal expression, but rather enhanced contrast for qualitative comparison. In addition, we provide the original WB image with marker.

We sincerely apologize for our oversight in failing to address this point in the previous revision. In the revised manuscript we have now included molecular-weight markers (kDa) on every Western blot panel and also the Original image.

Conclusions

-Are the conclusions supported by the data presented?

-Are the limitations of analysis clearly described?

-Do the authors discuss how these data can be helpful to advance our understanding of the topic under study?

-Is public health relevance addressed?

Reviewer #1: (No Response)

Reviewer #2: Please see Summary and General Comments

Reviewer #3: It is surprising to see that the authors did not cite the paper that discover the role of PIM1 in Toxoplasma infection. PIM1 controls GBP1 activity to limit self-damage and to guard against pathogen infection” (Science, 2023) by Frickel et al. The discussion should be robust and unbiased. Authors are encouraged to discuss (similarities/differences) with this study to make the discussion more comprehensive.

Response: We sincerely apologize for this oversight and the paper of Frickel et al. (Science, 2023) has now been added and fully discussed in the revised manuscript. In the updated Discussion (lines 561 to 577), we provide a detailed comparison between their findings and our results.

Editorial and Data Presentation Modifications?

Use this section for editorial suggestions as well as relatively minor modifications of existing data that would enhance clarity. If the only modifications needed are minor and/or editorial, you may wish to recommend “Minor Revision” or “Accept”.

Reviewer #1: (No Response)

Reviewer #2: Please see Summary and General Comments

Reviewer #3: (No Response)

Summary and General Comments

Use this section to provide overall comments, discuss strengths/weaknesses of the study, novelty, significance, general execution and scholarship. You may also include additional comments for the author, including concerns about dual publication, research ethics, or publication ethics. If requesting major revision, please articulate the new experiments that are needed.

Reviewer #1: The authors have adequately addressed the concerns of this reviewer in this revision.

Reviewer #2: Dear authors,

The manuscript has improved considerably after review; it is now clearer, more instructive, and more likely to be read and cited. I would like to congratulate you on the care with which you addressed the previous suggestions. Nevertheless, I still have a few specific suggestions that will bring greater clarity and precision of information to the work, particularly for researchers working on toxoplasmosis, in both experimental and population-based studies.

I suggest:

1-To include in the abstract that findings are limited to the acute phase of infection, underscoring the need for further studies in chronic-phase models.

2-To change the Introduction (lines 59-61): The statement ‘given the increasing drug resistance’ may overstate the current evidence. Clinical resistance in T. gondii is still rare, with only isolated reports and in-vitro studies demonstrating selectable resistance. I recommend rephrasing to emphasize the toxicity of current drugs and the potential (rather than established increase) for resistance, unless additional references are provided to support the claim of increasing resistance.

3-To change the Introduction (lines 73-75): I recommend replacing ‘atypical’ with ‘non-archetypal’. The term ‘atypical’ reflects only the historical order in which lineages I–III were first described and can imply that other lineages are rare exceptions, which is not the case in the regions mentioned. ‘Non-archetypal’ is more neutral, widely used in recent literature, and better conveys that these isolates represent the true breadth of the species’ genetic diversity, now grouped into multiple haplogroups (e.g., HG4–HG16). This change improves both accuracy and clarity without altering the original meaning.

4-To improve conciseness by splitting the overly long sentence in lines 513-518, as well as the one in 536-539, and by deleting repeated phrases such as “parasite within its host cells” and the duplicate mention of “PIM1 inhibitors.”

5-For better cohesion, add a short transition that links the main results to their clinical implications.

6-In the GBP1 paragraph, state explicitly how this pathway connects to your central finding on PIM1.

7-Methodologically, since the Western-blot data for the RH and Wh6 strains appear only in the Supplementary Material, include a brief quantitative summary in the Discussion (e.g., proliferation increased A–B ×).

8-Finally, address language issues: change the unnecessary capital letter in “Our study” (line 531), insert a hyphen in “proliferation-enhancing effects” (line 538), and replace “Noteworthy,” with “Importantly,” or “Of note,” (line 544).

Response: We thank the reviewer for the positive assessment of our revised manuscript and for the additional suggestions that further refine the clarity and accuracy of our work. Below we detail the changes made in response to each point.

1. Acute-phase limitation in Abstract

In the revised version (lines 13 to 15), the Abstract now ends with: "Since the role of PIM1 in chronic infection remains unexplored, follow-up studies using chronic models are essential. Collectively, our findings illuminate host–parasite interplay during acute toxoplasmosis and position PIM1 as a promising target for anti-*T. gondii* therapeutics."

2. Drug-resistance statement in Introduction

In the revised version (lines 59 to 64), the sentence now reads:Throughout this extended course, the regimen carries notable toxicities—leukopenia, thrombocytopenia, cutaneous rash, and fever—and, although clinically significant resistance remains rare, laboratory selection of dihydrofolate-reductase or -synthase mutations that raise the MIC has been readily achieved, underscoring the urgent need for safer, more effective anti-*T. gondii* drugs and vaccines. We removed the over-statement and cited recent reviews that underscore the rarity of clinical resistance.

3. Atypical” → “non-archetypal”

We replaced“atypical genotypes”with “non-archetypal genotypes” in lines 77.

4. Over-long sentences and repetitions

The sentences at lines have been split, and redundant phrases ("parasite within its host cells", duplicate "PIM1 inhibitors") have been removed.

5.A linking sentence was inserted at the start of the Clinical Implications sub-section:

In lines 551 to 553, a brief transitional sentence has been added after the cross-genotype data: "Taken together, these cross-genotype data provide a pre-clinical rationale for advancing PIM1-targeted interventions toward in-vivo validation and, ultimately, clinical evaluation."

6.GBP1 paragraph – explicit connection

We explicitly state (lines 561-577) how this pathway connects to my central finding on PIM1.

7.Quantitative summary for RH & Wh6 Western blots

We have followed your suggestion and added the quantitative summary. Please see lines 539 to 543.

8.Language micro-edits

We have followed your suggestion and added the quantitative summary:“Our study” to“our study” (line 544);“proliferation-enhancing effects”now hyphenated;“Noteworthy,” replaced with “Importantly,” (line 558).

Reviewer #3: Stated above

PLOS authors have the option to publish the peer review history of their article (what does this mean?). If published, this will include your full peer review and any attached files.

If you choose “no”, your identity will remain anonymous but your review may still be made public.

Do you want your identity to be public for this peer review? For information about this choice, including consent withdrawal, please see our Privacy Policy.

Reviewer #1: No

Reviewer #2: No

Reviewer #3: No
